# Selection, drift and community interactions shape microbial biogeographic patterns in the Pacific Ocean

**DOI:** 10.1038/s41396-022-01318-4

**Published:** 2022-09-17

**Authors:** Felix Milke, Irene Wagner-Doebler, Gerrit Wienhausen, Meinhard Simon

**Affiliations:** 1 Institute for Chemistry and Biology of the Marine Environment, University of Oldenburg, Carl von Ossietzky Str. 9-11, D-26129 Oldenburg, Germany; 2 Institute of Microbiology, Technical University of Braunschweig, D-38106 Braunschweig, Germany; 3 Helmholtz Institute for Functional Marine Biodiversity at the University of Oldenburg (HIFMB), Ammerländer Heerstraße 231, D-26129 Oldenburg, Germany

**Keywords:** Microbial ecology, Microbiology

## Abstract

Despite accumulating data on microbial biogeographic patterns in terrestrial and aquatic environments, we still lack a comprehensive understanding of how these patterns establish, in particular in ocean basins. Here we show the relative significance of the ecological mechanisms selection, dispersal and drift for shaping the composition of microbial communities in the Pacific Ocean over a transect of 12,400 km between subantarctic and subarctic regions. In the epipelagic, homogeneous selection contributes 50–60% and drift least to the three mechanism for the assembly of prokaryotic communities whereas in the upper mesopelagic, drift is relatively most important for the particle-associated subcommunities. Temperature is important for the relative significance of homogeneous selection and dispersal limitation for community assembly. The relative significance of both mechanisms was inverted with increasing temperature difference along the transect. For eukaryotes >8 µm, homogeneous selection is also the most important mechanisms at two epipelagic depths whereas at all other depths drift is predominant. As species interactions are essential for structuring microbial communities we further analyzed co-occurrence-based community metrics to assess biogeographic patterns over the transect. These interaction-adjusted indices explained much better variations in microbial community composition as a function of abiotic and biotic variables than compositional or phylogenetic distance measures like Bray–Curtis or UniFrac. Our analyses are important to better understand assembly processes of microbial communities in the upper layers of the largest ocean and how they adapt to effectively perform in global biogeochemical processes. Similar principles presumably act upon microbial community assembly in other ocean basins.

## Introduction

Biogeography of microbes is a young field compared to biogeography of plants and animals, which has been studied for more than a century [1, 2]. Microbial biogeographic patterns have been established for terrestrial [3–5] as well as aquatic systems from regional to global scale [6–13]. Selection, dispersal, drift and speciation are the major ecological mechanisms leading to species sorting and community assembly [14, 15]. Despite accumulating reports of biogeographic patterns of microbial communities, we still lack a comprehensive understanding of the relative significance of these mechanisms for shaping such patterns, in particular in basin-scale oceanic regions [15–19]. Several studies assessed in such regions taxonomic distance-decay relationships of microbial communities which reflect the net result of selection, dispersal and drift [9, 11, 13, 20–22]. The phylogenetic distance among microbial communities of different regions can also be used to assess biogeographic patterns and thus distance-decay patterns of phylogenetically related clusters [23]. Recently, taxonomic (TINA) and phylogenetic interaction-adjusted indices (PINA) were introduced to analyze the variance of microbial community structure as a function of environmental variables [18]. In contrast to compositional and phylogenetic distance measures these indices consider potential species interactions, based on co-occurrence network analyses, in biogeographic assessments. Only one study tested the relative significance of ecological mechanisms for shaping biogeographic patterns and environmental effects on interaction networks of pro- and eukaryotic picoplankton communities [24]. This study, focusing on tropical and subtropical sunlit oceanic regions of the global data sets of the Malaspina expedition, showed that picoeukaryotic communities were predominantly structured by dispersal limitation and prokaryotic communities by the combined action of dispersal limitation, selection and drift. Further, temperature-driven selection appeared as a major mechanism affecting prokaryotic species co-occurrence. To understand how ocean basin-wide biogeographic patterns of microbial communities establish and persist, all representative regions of an ocean basin need to be covered in such an analysis. As there is limited but important exchange of epipelagic and mesopelagic waters and thus of planktonic microbes by up- and downwelling in mesoscale eddies [25], equatorial upwelling [12] and seasonal overturn [26, 27] it is important to include both layers in such an analysis, also because epipelagic microbes associated to sinking particles may be introduced into and proliferate in the mesopelagic [28].

In order to complement our understanding of how marine microorganisms assemble into diverse communities along environmental gradients, we aimed at disentangling the significance of ecological mechanisms for shaping biogeographic patterns of microbial communities in the epi- and upper mesopelagic layers of the Pacific Ocean between subantarctic and subarctic regions. Elucidating the baselines for establishing biogeographic microbial patterns in the globally largest ocean basin with widely differing latitudinal hydrographic and nutrient regimes will greatly help better understand how pelagic microbial communities evolve, establish and adapt to changing environmental conditions. Hence, we analyzed the relative significance of selection, dispersal, drift and of interaction-adjusted indices TINA and PINA relative to classical compositional and phylogenetic distance measures to explain microbial biogeographic patterns and their variations as a function of environmental variables in this ocean. The basis were amplicon sequence variants (ASV) of the 16S and 18S rRNA genes of samples collected at 26 stations between 20 and 500 m depth between the subantarctic and subarctic Pacific along a 12,400 km transect, covering a large temperature, nutrient and productivity gradient from equatorial to subpolar regions. Speciation as an ecological mechanism was not considered because it would require long-term repetitive sampling of the same community members in the same water body. Our analyses are embedded in a comprehensive assessment of hydrographic, biogeochemical and microbial features along this transect [29, 30].

## Materials and methods

### Rationale of the study, sampling and biogeographic provinces

**Fig. 1:**
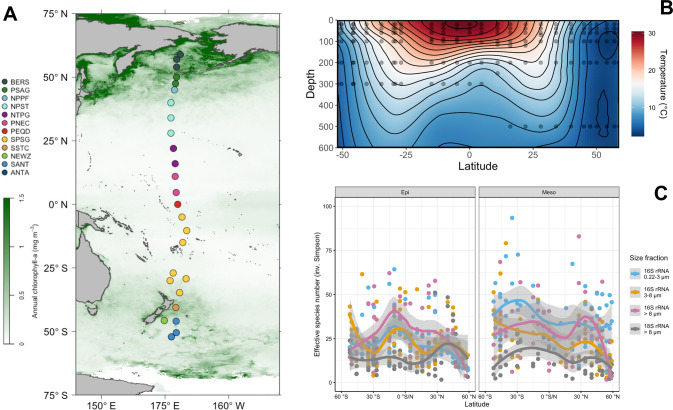
Sampling stations, water temperature and microbial richness in the epi- and mesopelagic between subantarctic and subarctic waters of the Pacific Ocean. **A** Stations between 52°S and 59°N. The colour code of the stations refers to their affiliation to biogeographic provinces according to Longurst [32]. BERS Bering Sea, PSAG Pacific subarctic gyre, NPPF north Pacific polar frontal region, NPST north Pacific subtropical gyre, NPTG north Pacific tropical gyre, PNEQ Pacific north equatorial counter current, PEQD Pacific equatorial divergence, SPSG south Pacific subtropical gyre, SSTC south subtropical convergence; NEWZ, New Zealand coastal province, SANT subantarctic province. Stations are overlaid on a map of the Pacific Ocean with annual mean concentrations of chlorophyll *a* at the surface (https://oceandata.sci.gsfc.nasa.gov). **B** Contour graph of water temperature along the transect based on continuous measurements by a temperature probe at each station between the surface and 600 m depth. Black dots indicate the depth of sampling. **C** Effective number of species (here ASV; inverse Simpson index) richness of the free-living (0.2–3 µm), small (3–8 µm) and large particle-associated (>8 µm) prokaryotic and eukaryotic microbial communities at the stations in epipelagic (20–100 m) and mesopelagic waters (200–500 m) at the stations along the transect. Single data points at the depths and stations and loess fit and its 95% confidence interval for the epi- and mesopelagic are given.

The study intended to assess microbial communities in the epi- and mesopelagic Pacific Ocean from subantarctic to subarctic waters and thus in all biogeographic provinces along this transect. Therefore, samples were collected aboard RV *Sonne* in the Pacific Ocean during two cruises at 26 stations along a transect closely following 180° longitude E/W between 52.1°S southeast of New Zealand and 58.9°N in the Bering Sea (Fig. 1A and Supplementary Table S1). The first cruise (SO248) encompassed stations 1–19, started in Auckland, New Zealand, on May 1st and ended in Dutch Harbour, Alaska, USA, on June 3, 2016. The second cruise (SO254; Auckland–Auckland) took place during the austral summer from January 26th to February 27, 2017, and covered the southernmost stations 20–26 of the transect. Samples were collected at all stations at the following depths: 20, 40, 60, 100, variable depth of the deep chlorophyll maximum, 200, 300 and 500 m. Sampling was carried out using 20 L-Niskin bottles mounted on a Sea-Bird Electronics 32 Carousel Water Sampler containing 24 × 20 L-Niskin bottles (Ocean Test Equipment Inc., Ft. Lauderale, FL, USA). The carousel included the CTD system SBE 911 plus a probe with double-sensors for temperature (SBE 3), conductivity (SBE 4), pressure (Digiquartz), Chl *a*-fluorescence combined with turbidity (FluoroWetlab ECO_AFL FL, WET Labs Inc., Bellevue, WA, USA), dissolved oxygen (Optode 4831F, Aanderaa, Bergen, Norway) and an altimeter (Teledyne Benthos, North Falmouth, MA, USA). After retrieval, 12 L of sample water from one bottle was transferred to 20 L wide-mouth barrels and filtered sequentially through membrane filters of the following pore sizes: 8 µm (mixed cellulose ester SCWP14250, Millipore, Darmstadt, Germany), 3 µm (mixed cellulose ester SSWP14250, Millipore) and 0.22 µm (polyethersulfone GPWP14250, Millipore). After filtration membranes were immediately stored at −80 °C until DNA extraction. For further details on sampling and the filtration procedure see Milici et al. [31] and Giebel et al. [30]. Biogeographic provinces according to Longhurst [32] were determined based on measured environmental parameters as described in Giebel et al. [30].

### DNA extraction and library preparation with mock communities

We used the PowerSoil kit from QIAGEN (Hilden, Germany) formerly known as Powersoil Kit (MoBio Laboratories) to extract total DNA from the filters. 1/8 of the frozen filters were used and cut into small pieces before transferring it directly into the PowerSoil lysis solution including garnet beads from the kit. Extraction procedure was done according to the manufacturer, except for some modifications after Milici et al. [31]. In short, bead beating was extended up to one hour and afterwards an additional lysis step using a proteinase K (22 mg/ml) incubation at 37 °C was added.

Total DNA was amplified using universal V4-V5 primers [33] which target 16S and 18S rRNA genes. Using these primers, we were able to recover bacterial and archaeal sequences within one sample and thereby infer their relative community proportions. The V4-V5 primers also amplify 18S rRNA gene sequences that were used to assess the eukaryotic communities as done in a previous study [34]. Taxonomic coverage of the amplified 18S rRNA fragment was assessed using the tool pr2-primers [35]. The library preparation was done according to Yeh et al. [36] using primers that included barcoded indices and Illumina adaptors. Doing so, we only used one PCR step with 30 cycles instead of two PCR steps that typically result in more than 30 total cycles and thereby recover a higher sequence diversity. We used magnetic beads for PCR clean-up and size fractioning (AMPureXP beads in 0.8X ratio, Beckman Coulter, Brea, CA, USA). Cleaned amplicons were diluted to equal DNA concentrations measured by Qubit (ThermoFisher, Waltham, MA, USA) and up to 96 samples were pooled together for sequencing. The sample pool was sequenced on a MiSeq (Illumina, San Diego, CA, USA) in PE300 mode. Samples with <20,000 reads were re-sequenced to ensure adequate sequencing depth for every sample and samples with <8000 reads after bioinformatics processing were excluded from further analyses.

Four different mock communities were added to each sequencing pool to validate the sequencing run and confirm primer coverage for all included taxa. We used the same four mock communities as described in Parada et al. [33], namely 16S even & staggered and 18S even & staggered mocks. After sequencing and bioinformatic processing of all sequences, inferred mock communities were compared to their true community proportions.

### Bioinformatic pipeline

Samples were demultiplexed at the sequencing facility according to their respective barcoded indices in the forward and reverse reads. Subsequently, sequences were processed using the bioinformatic pipeline described in Yeh et al. [36] using QIIME2 as a wrapper for the DADA2 denoising algorithm. Briefly, primer sequences were removed using cutadapt (version 3.2) allowing a mismatch of 20% within primer sequences. Reads were split into a 16S and 18S sample set using the bbtools package (version February 2017) and a curated database derived from SILVA132 [37] and PR2 (version 4.12.0) [38]. The isolated 16S rRNA gene sequences were further analyzed using QIIME2 (version 2019.4) [39]. We first checked read quality and cut all forward reads at 250 bp and reverse reads at 220 bp to remove low-quality ends. Subsequently, the QIIME2 plugin q2-dada2 [40] was used to denoise the sequences with the DADA2 denoising algorithm, including merging of sequences and removal of chimeric reads. Finally, we run the qiime2 classify-sklearn plugin for classification of sequences against the SILVA132 database which was subset to the V4-V5 primer region. All sequences assigned as chloroplasts were removed for downstream analyses. 18S rRNA gene sequences were treated similarly, applying the same analysis pipeline in QIIME2 but using the PR2 database for classification.

### Abundance filter

We used an abundance-filter to remove possibly ambiguous and very rare sequences such as singletons from the dataset. The filter included only ASVs that were present in an abundance >0.001% of the complete dataset and (i) in two or more samples at a relative abundance of >1%, or (ii) in ≥2% of all samples in an abundance of >0.1%, or (iii) in at least 5% of all samples in any abundance [31]. Its implications on biogeographic analyses were analyzed in a previous study [34].

### Compositional and phylogenetic distance-decay analyses

Geographic distance decay analyses were based on either Bray–Curtis distances to display compositional turnover or TINA distances for the turnover of interacting organisms. Geographic distance was calculated using the Haversine formula.

TINA and PINA distances were calculated according to Schmidt et al. [18]. The underlying co-occurrence structure was inferred using SparCC [41]. Both indices were implemented using a modified version of the scripts from Schmidt et al. (https://github.com/defleury).

For phylogenetic distance-decay analyses we computed unweighted UniFrac distances between subsets of communities containing only individual taxonomic ranks of families. Those families were chosen based on two conditions: (i) families with more than 10 different ASVs, (ii) families that were found in at least 10,000 km sequentially along the transect. We aligned sequences of all ASVs of the respective family to a random family member for UniFrac distances, using the ClustalW multiple sequence alignment algorithm from the *msa* package [42]. We included only sample combinations from the same size fraction, the same depth group and with an overlap of ASVs between >0% and <100% for distance-decay analysis. To group all resulting distance-decay graphs into clusters of similar patterns, we normalized UniFrac distances using z-scoring, which helped to avoid clustering of graphs based on mean dissimilarity differences. Next, we computed a loess model for each family that fitted the average distance-decay pattern. We decided for non-parametric local regression fitting instead of parametric models to account for different curve patterns and increase detection sensitivity of clusters. For clustering, we predicted the UniFrac distance at equidistant locations along the transect using the loess model, resulting in a UniFrac x location matrix. Based on that, we inferred numbers of cluster using the average silhouette method with hierarchical clustering [43].

### Phylogenetic tree construction

Global phylogenetic trees were inferred by first aligning all sequences present in the dataset against the SILVA 132 alignment using *SINA* (version 1.2.9) [44]. Next, separate trees for prokaryotic and eukaryotic alignments were inferred using FastTree (version 2.1.11) [45]. Phylogenetic distances were calculated using the cophenetic function from the stats package.

### Identification of phylogenetic signal

We analyzed the phylogenetic signal of microbial communities to measure if selection impacts phylogenetic differentiation within our dataset [46, 47]. To do so, we first compared phylogenetic dissimilarities and habitat differences between microbial communities [17, 24] and additionally, we calculated habitat preferences of each ASV individually and compared habitat difference and phylogenetic dissimilarity for each pair of ASVs [47]. We used the abundance-weighted βMNTD (beta-mean-nearest-taxon-distance) as phylogenetic dissimilarity metric for community-wise comparison (see below) and temperature to define environmental conditions as the parameter that explained the highest proportion of community variance. The presence of phylogenetic signal was analyzed using a Mantel-correlogram with 999 permutations. The phylogenetic dissimilarities were binned into 40 bins.

To infer phylogenetic signal on the level of ASVs, we defined an ecological niche for each ASV using a set of different environmental parameters that were chosen by having a large impact on community composition. The niche of an ASV was defined as the weighted mean of measurements of variables using relative ASV abundances as weights. We did this for temperature, NO_*x*_ and oxygen and additionally for the first two principal component axes of a PCA involving a larger set of environmental parameters. We used cophenetic distances as measure of phylogenetic dissimilarity and binned it into 100 equally sized dissimilarity groups.

### Quantification of ecological mechanisms

We quantified the ecological mechanisms that shaped microbial community composition after Stegen et al. [17]. This framework relies on the principle that ecologically similar organisms share a similar phylogeny and that a significant phylogenetic turnover between two communities expresses a selection process. Further, if no significant phylogenetic effect is found, a significant compositional turnover between two samples is interpreted as a dispersal process. Both processes may be homogenizing by promoting a significant similarity between two samples or heterogenizing, which promotes a significant difference (i.e., dispersal limitation or heterogeneous selection). If no significant phylogenetic or compositional effect is found between two samples, a dominance of neutral effects is assumed (ecological drift). Phylogenetic distance was inferred by first aligning all sequences and building a tree as written above and then calculating cophenetic distances of the tree.

The phylogenetic similarity is calculated as βMNTD [48] which quantifies the average phylogenetic distance between each ASV and its phylogenetically most related ASV in another sample. A null model is acquired by shuffling species names and abundances and calculating the βMNTD for 999 shuffled datasets. The deviance of the mean of all shuffled βMNTD indices by >+2 or <−2 standard deviations is interpreted as a significant turnover with >+2 indicating heterogeneous selection and <−2 indicating homogeneous selection.

A compositional turnover is quantified by the Raup-Crick index adjusted for relative abundances [49]. This involves probabilistic re-assembly of local communities by maintaining observed richness and number of individuals. In the reassembly of communities, the probability of observing an ASV count was related to the number of samples the ASV was detected in and its relative abundance in all samples. Each community pair is reassembled 999 times and a Bray–Curtis dissimilarity is calculated to quantify compositional turnover for each re-assembly, which is used as a null distribution. The difference between observed Bray–Curtis and its null distribution is scaled from −1 to +1, hence defined as RC_bray_ [17]. An RC_bray_ > +0.95 is defined as a dispersal limitation and an RC_bray_ < −0.95 indicates homogenizing dispersal. Ecological drift is inferred if no significant phylogenetic or compositional turnover could be detected, therefore defined as |βMNTD| < 2 and |RC_bray_| < 0.95.

### Statistics

All statistical analyses were performed with R (R Core Team 2021, version 3.6.1). To improve handling of ecological data types (count matrix & environmental data), we invented wrapper functions for some of the main dplyr-package principles on data wrangling (see https://github.com/dermilke/ExCom). This greatly improved computational efficiency and overall productivity of statistical analyses. R scripts for analysis pipelines are accessible via GitHub (https://github.com/dermilke/Pacific_Bacterioplankton). We used satellite data for sea surface chlorophyll concentrations (annual mean, 4′ resolution) from MODIS-Aqua [50] to display the sampling region. The corresponding map was produced using the oceanmap package (version 0.1.3). Depth sections of selected environmental parameters were created using the high depth-resolution CTD dataset by interpolating the space between samples using local polynomial regression fitting. The vegan package (version 2.5.7) was used for calculating the inverse Simpson index (effective number of species, here ASVs), Bray–Curtis distances, non-metric multidimensional scaling (NMDS) ordination and ANOSIM analyses. Regarding the bar plots, we assigned all respective taxonomic ranks with an abundance below 0.8% of the complete dataset to the “Others” group for visualization purposes. Before calculating NMDS, count data was normalized by their total sum and square-root transformed before converting them into Bray–Curtis distances. Prior to alpha-diversity calculation we rarefied all samples to equal sampling depth of 8000 counts per sample which was chosen as robust threshold that allowed for sufficient sequencing depth while not losing too many samples due to low sequencing depth.

To show SAR11 Clade I oligotype abundances along the transect, we normalized abundances for each ASV across the transect. Z-score normalization was used for this purpose. Normalized abundances of analyzed taxonomic groups were displayed in a heatmap whose lines (ASVs) were arranged by hierarchical clustering.

PERMANOVA analyses were conducted using the adonis function from the vegan package. Therefore, a subset of environmental variables was selected and we individually tested their impact on community variation. We ran 999 permutations and dropped parameters whose Benjamini–Hochberg corrected *p* values exceeded 0.05. This analysis was run with four different distance measures: Bray–Curtis distances, TINA, PINA and UniFrac. The PERMANOVA was run for each size fraction individually.

## Results

Our transect covered all biogeographic provinces [32] from the subantarctic Pacific to the Bering Sea (Fig. 1A). The two cruises were scheduled such that we visited the stations in the northern temperate and subarctic region in May and those in the austral temperate to subantarctic region in late January and February thus covering these regions of both hemispheres in summer. In the (sub)tropical provinces between 40°S and 30°N, water temperatures ranged from 22–30 °C in the upper 60 m, and beyond 45°N and 50°S they remained below 10 °C (Fig. 1B). Highest concentrations of chlorophyll *a* occurred in the temperate to subpolar regions and lowest concentrations in the warm regions (Supplementary Fig. S1B). Concentrations of inorganic nutrients and particulate organic carbon and nitrogen and prokaryotic cell numbers exhibited similar patterns as chlorophyll *a*. For salinity see Supplementary Fig. S1A and for the other variables references [29, 30]. Samples from 20, 40, 60 and 100 m depth were used to assess epipelagic and from 200 and either 300 or 500 m to assess mesopelagic microbial communities. Five hundred m were sampled from 15° to 59°N whereas 300 m between 52°S and 10°N. Sampling of these different depths did not affect our analysis in the mesopelagic as similarity values of the microbial community composition at 200 and 300 versus 200 and 500 m depth were not significantly different (Wilcoxon *p* value = 0.23). For a more refined analysis we differentiated among the free-living (0.2–3 µm), small (3–8 µm) and large (>8 µm) particle-associated (PA) microbial subcommunities. From the 482 samples collected, we retrieved 11,019,436 ASV reads with a mean sequencing depth of 22,600 prokaryotic reads per sample and 7147 eukaryotic reads. Bacterial, archaeal and eukaryotic reads comprised 76%, 7% and 17%, respectively (Table S1). Because of the very low coverage, eukaryotes were not assessed in the 0.2–3 µm and 3–8 µm size fractions.

The effective number of species (here of ASVs) of the epipelagic prokaryotic communities in all three size fractions peaked in the tropics but varied greatly along the transect. Each size fraction exhibited distinct latitudinal patterns and the 0.2–3 µm and >8 µm size fractions lowest values in the northern- and southernmost regions (Fig. 1C). The 3–8 µm size fraction exhibited low values also in the subtropical gyres and highest values in the southernmost region. In the mesopelagic, the effective number of species of the 0.2–3 µm and 3–8 µm prokaryotic communities were highest in the southern hemisphere whereas that of the >8 µm prokaryotic communities peaked in the north Pacific subtropical gyre. The PA fractions exhibited lowest values in the northernmost regions. Effective numbers of microeukaryotic species were highest in the epipelagic in the northern temperate region but values were generally lower compared to prokaryotes (Fig. 1C). The low values were a result of the much lower evenness (data not shown). The pattern in the mesopelagic covaried with that of the >8 µm prokaryotic communities with a weaker increase in the northern subtropical region.

**Fig. 2:**
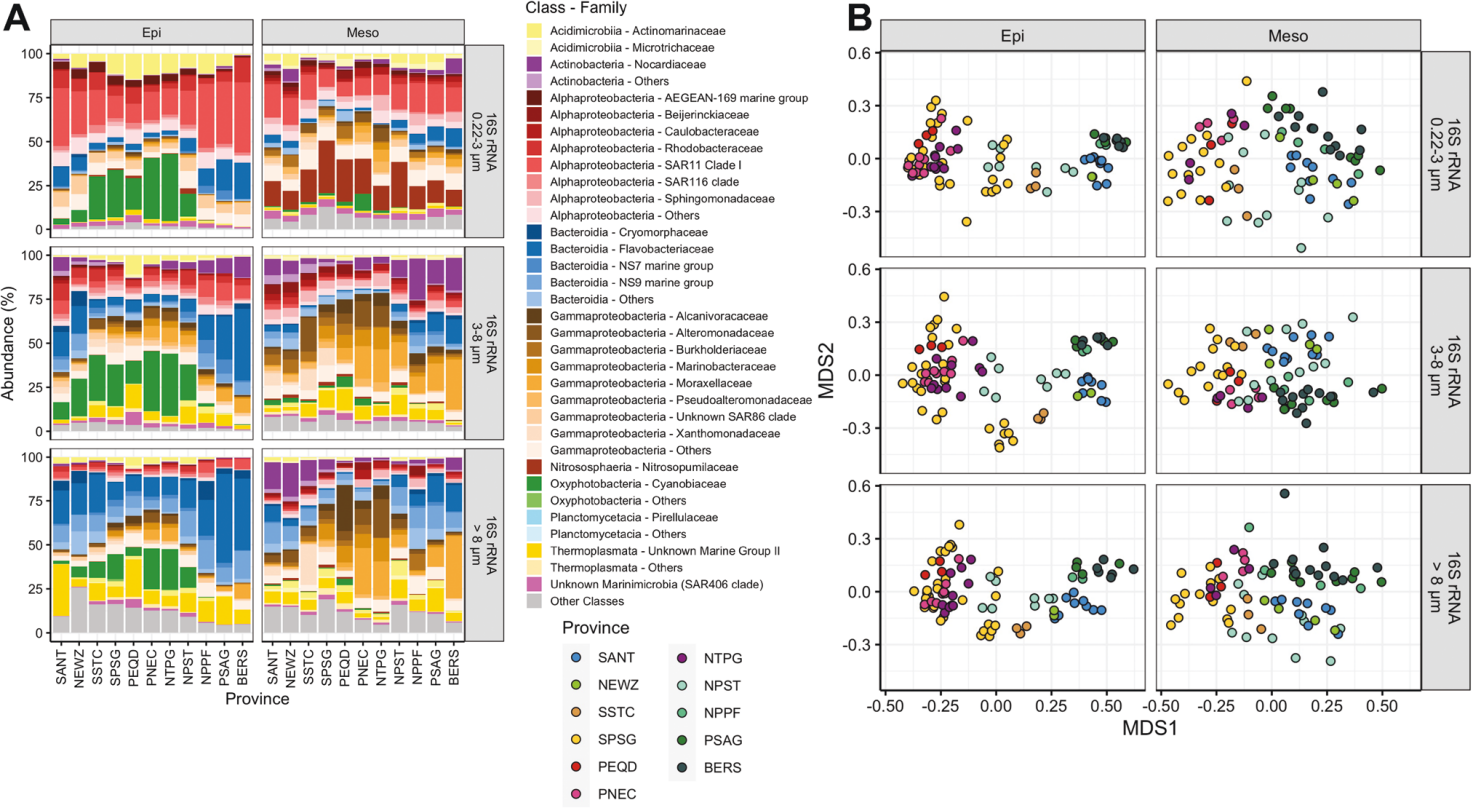
Phylogenetic composition and NMDS analysis of prokaryotic communities in the biogeographic provinces of the epi- and mesopelagic Pacific between subantarctic and subarctic regions. **A** Relative abundance (% of total) of prokaryotic classes and major families in each biogeographic province of 0.2–3 µm, 3–8 µm and >8 µm prokaryotic communities in the epi- and mesopelagic. Stacked bars of each province are means of all stations and depths of a given province and layer. “Others” include those which constitute <0.8% each of the respective taxonomic level. **B** NMDS analysis of the 0.2–3 µm, 3–8 µm and >8 µm prokaryotic communities of all stations and depths in the epi- and mesopelagic.

*Alphaproteobacteria*, *Oxyphotobacteria* and *Bacteroidia* constituted highest proportions of the prokaryotic communities in the epipelagic along the transect with variations predominantly between (sub)tropical, temperate and subpolar provinces and among the different size fractions (Fig. 2A). Other phyla such as *Acidimicrobia*, *Actinobacteria*, *Gammaproteobacteria* and *Thermoplasmata* were also detected but with lower proportions and a different partitioning in the three size fractions. In the mesopelagic, ammonium-oxidizing *Nitrososphaeria* comprised proportions of 10–30% in the 0.2–3 µm fraction along the transect and *Gammaproteobacteria* 10–60% in the PA fractions, with highest proportions in (sub)tropical provinces. In this layer *Alphaproteobacteria* and *Bacteroidea* encompassed generally lower proportions (Fig. 2A). In the eukaryotic communities *Dinophycea* and *Syndiniales* constituted the major phyla in the epipelagic but *Spirotrichea* and *Bacillariophyta* encompassed substantial proportions in the temperate to subpolar regions in both hemispheres (Supplementary Fig. S2). In the mesopelagic, other phyla contributed relatively higher proportions such as *Acantharea* in the SPSG and PNEQ and *Cephaloidophoroidea* in the temperate to subpolar southern hemisphere (Supplementary Fig. S2). We tested the taxonomic coverage of the universal primer pair we applied against the PR2 database using the pr2-primers tool allowing two mismatches [35]. The applied primer amplified 97.08% of all taxonomic groups within the PR2 database.

The composition of the prokaryotic communities among the different size fractions, the epi- and mesopelagic depths and provinces was significantly different as tested by an ANOSIM (size fractions: *p* < 0.001, *r* = 0.21; Depth: *p* < 0.001, *r* = 0.25; Province: *p* < 0.001, *r* = 0.31). An NMDS analysis revealed a systematic shift of the community composition in all size fractions from subpolar to tropical regions, separating communities according to temperature regimes (Fig. 2B). The (sub-)tropical warm provinces (SPSG, PEQD, PNEC, NTPG) were clearly separated from subtropical/temperate (SSTC, NPST) and subpolar provinces (SANT, NEWZ, NPPF, PSAG, BERS). In the epipelagic, different regions exhibited more distinct clusters than in the mesopelagic, reflecting the stronger separation and more pronounced provincialism of water masses in the epipelagic. Ordination axis MDS1 was highly anticorrelated with temperature (size fractions epipelagic: cor. *p* < 10^−36^, Pearson’s *r* < −0.928; mesopelagic: cor. *p* < 10^−11^, −0.75 ≤ Pearson’s *r* ≤ −0.68).

### Distance decay patterns

**Fig. 3:**
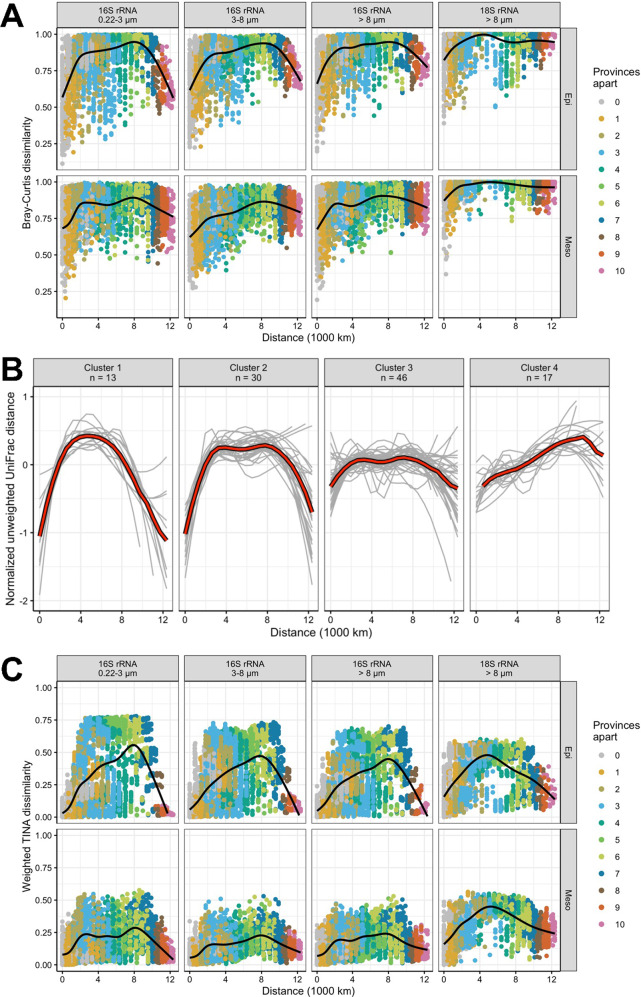
Dissimilarities of prokaryotic communities and taxonomic groups captured by different β-diversity metrics related to geographic distance between the subantarctic and subarctic Pacific. **A** Compositional dissimilarity (Bray–Curtis) against geographic distance of the 0.2–3 µm, 3–8 µm and >8 µm prokaryotic communities in the epi- and mesopelagic. Colours of points indicate number of biogeographical provinces between samples and black line shows loess-fit. **B** Phylogenetic dissimilarity (UniFrac) of members within taxonomic families as a function of geographic distance grouped into clusters 1 to 4. Grey lines indicate single families and the red line means of the respective cluster. **C** TINA dissimilarity against geographic distance of the 0.2–3 µm, 3–8 µm and >8 µm prokaryotic communities in the epi- and mesopelagic. The black line represents a loess fit for each subset.

To analyze species turnover and community composition as a function of geographic and phylogenetic distance and temperature and as a net effect of the ecological mechanisms and co-occurrence structure we assessed Bray–Curtis dissimilarity, unweighted UniFrac phylogenetic dissimilarity and the TINA index in our data set. The Bray–Curtis dissimilarity patterns of prokaryotic communities in all size fractions in the epipelagic exhibited a bell-shaped pattern with an initial steep and a slight further increase or plateau at intermediate distances and a final steep decrease returning to initial dissimilarities (Fig. 3A). As the largest distances represent the subpolar provinces these patterns indicate a higher similarity between communities in these provinces with rather similar environmental conditions. These patterns were most pronounced in the 0.2–3 µm size fraction. Dissimilarities ranged between 60 and 90%, with increasing percentages at the shortest and largest distances from the 0.2–3 µm to the >8 µm fraction. In the mesopelagic, patterns were basically similar but dissimilarity ranges along the distance lower than in the epipelagic. In the eukaryotic communities, dissimilarities were generally greater than in the prokaryotic communities, exceeding 80% as the mean for all stations and depths (Fig. 3A).

We further assessed phylogenetic distance decay patterns of prokaryotes and included 106 phylogenetic families. The unweighted UniFrac metric was applied to account for all taxa detected irrespective of their relative abundance. We identified by silhouette clustering four distinct clusters differing in their latitudinal patterns (Fig. 3B, Supplementary Fig. S3 and Supplementary Table S2). Cluster 1, encompassing 13 families and including SAR11 Clade I and *Cyanobiaceae*, was most abundant in the epipelagic 0.2–3 µm fraction. Its phylogenetic distance decay pattern was bell-shaped with the highest dissimilarity from 4000–6000 km and strongly decreasing dissimilarities at shorter and larger distances. Cluster 2, encompassing 30 families, was relatively prominent in the epipelagic and mesopelagic 0.2–3 µm fractions and included *Rhodobacteraceae*, three *Flavobacterales* families and *Nitrosopumilaceae* as some of the most abundant families. This cluster exhibited also a bell-shaped pattern with highest dissimilarities from 3000–8000 km and strongly decreasing dissimilarities at shorter and larger distances but with a smaller dissimilarity range than cluster 1. Cluster 3, the largest cluster with 46 families, was most prominent in the mesopelagic, in particular in the PA fractions, and varied greatly along the transect. It included *Alteromonadaceae* and other gammaproteobacterial families. This cluster exhibited only small changes along the entire distance range. Members of cluster 4 with 17 families were generally least abundant along the transect. The dissimilarity of this cluster continuously increased to a distance of 10,000 km.

To also consider community variance by potential interactions among community members based on co-occurrences [18] we analyzed distance decay patterns of the TINA index of the microbial communities. This analysis yielded rather similar and roof-shaped patterns, slightly skewed to larger distances, for all prokaryotic size fractions in the epipelagic with a dissimilarity of <10% at shortest and largest distances (Fig. 3C). Highest dissimilarities of 45–55% occurred at distances of 8000 km with slightly lower values in the 3–8 µm and >8 µm fractions. The distance decay pattern of microeukaryotes was skewed to smaller distances with a dissimilarity peak of 50% between communities at distances of 5000 km in the epi- as well as mesopelagic. We also assessed the TINA index as a function of temperature difference. It was saturated for prokaryotic communities in the epi- and mesopelagic at temperature differences of 20° and 15 °C, respectively (Supplementary Fig. S4). In the epipelagic, dissimilarity increased from below 10% at temperature differences of <5 °C in all size fractions to 70% at differences of 20 °C in the 0.2–3 µm fraction and to 60% in the 3–8 µm and >8 µm fractions. In the mesopelagic dissimilarities remained consistently lower. The same analysis for microeukaryotic communities indicated a generally greater TINA distance than for prokaryotic communities, saturating above 20 °C in the epipelagic but not in the mesopelagic (Supplementary Fig. S4). It is noteworthy that unlike for prokaryotes and indicated by the large range of individual data points at low-temperature difference, eukaryotic communities with negligible temperature differences could express high TINA dissimilarities in the epipelagic.

### Population patterns of the SAR11 clade along the transect

**Fig. 4:**
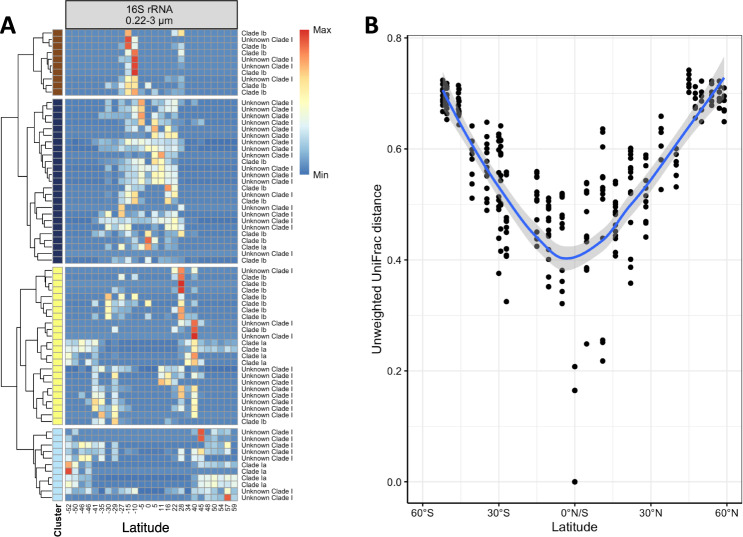
Latitudinal patterns and phylogenetic distance of ASVs of the SAR11 Clade 1 along the transect. **A** Heatmap of abundances of SAR11 Clade I-ASVs in the epipelagic along the transect. Abundance was normalized row-wise and displayed by colour. Classification of each ASV is based on SILVA132. Rows are sorted by hierarchical clustering based on Bray–Curtis and separated by gaps that represent clusters inferred from silhouette clustering; **B** UniFrac dissimilarity of members of the SAR11 Clade I in the epipelagic along the transect. Black dots indicate SAR11 clade I community members of individual samples and the blue line and grey shaded area a loess-fit and its 95% confidence interval.

SAR11 Clade I was the single most abundant phylogenetic lineage along the transect (see above, Fig. 2A). Using ASVs enabled us to resolve 16S rRNA gene oligotypes and thus to test for the existence of distinct ecotype populations as a result of diversification of this lineage along the transect. These populations formed a V-shaped latitudinal pattern with distinct ASVs in equatorial regions and continuous transitions to other ASVs/ecotypes towards higher absolute latitudes (Fig. 4A). This resulted in completely different groups of SAR11 Clade I ecotypes between tropical and subpolar provinces but similar populations in the corresponding mid to high latitudes of both hemispheres with comparable environmental conditions. We tested whether the differential abundance of these populations along the transect was reflected also in a phylogenetic differentiation. Therefore, we calculated unweighted UniFrac-distances between these populations relative to that near the equator. We used this measure to focus on sequence similarity without taking into account sequence abundance, already considered in the relative abundances (Fig. 2A), and to emphasize the evolutionary relationship among SAR11 Clade I ecotypes. For the epipelagic, it yielded patterns that resembled the V-shaped distribution of ASVs, indicating an evolutionary divergence of SAR11 Clade I populations between tropical, temperate and subpolar regions (Fig. 4B). Patterns in the mesopelagic were similar in the temperate to subpolar regions but less pronounced in the subtropics and tropics (Supplementary Fig. S5).

### Ecological mechanisms and variables shaping biogeographic patterns

**Fig. 5:**
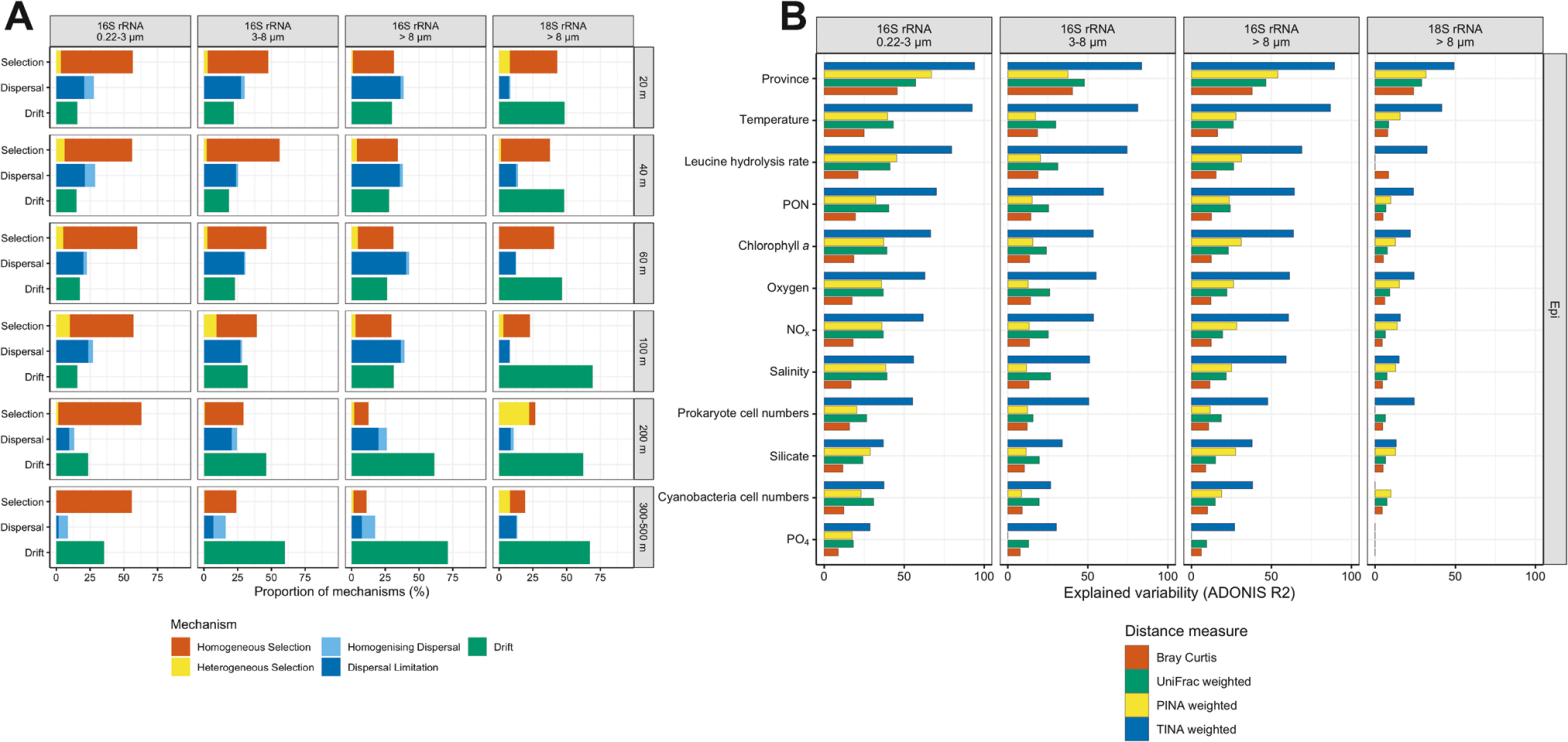
Ecological mechanisms shaping biogeographic patterns and environmental variables affecting the structure of epipelagic microbial communities as captured by different β-diversity metrics between the subantarctic and subarctic Pacific. **A** Relative contribution of homogeneous and heterogeneous selection, homogenizing dispersal and dispersal limitation and stochastic drift on community assembly of the 0.2–3 µm, 3–8 µm and >8 µm prokaryotic and 18S eukaryotic communities in the epipelagic (20, 40, 60, 100 m) and mesopelagic (200, 300 or 500 m). 300 m was sampled from 52°S to 10°N and 500 m from 15° to 59°N. **B** Community variance individually explained by single environmental variables for different dissimilarity indices (Bray–Curtis, UniFrac, TINA and PINA) for 0.2–3 µm, 3–8 µm and >8 µm prokaryotic communities in epipelagic depths. Explained variance was inferred from PERMANOVA and only significant results with Benjamini-Hochberg adjusted *p* values < 0.05 are dis*p*layed.

Bray–Curtis dissimilarity as a function of geographic distance revealed pronounced differences among the prokaryotic and eukaryotic communities between different distance sections, and these differences were more distinct in the epi- than in the mesopelagic. This finding implies that the ecological mechanisms selection, dispersal and drift acted differently upon the assembly of the prokaryotic and eukaryotic communities along the transect and in the epi- and mesopelagic. To analyze the effect of each mechanism as a proportion of all three mechanisms for community assembly along the transect we applied an established analytical framework based on data comparisons to null models [17]. Therefore, we first tested for phylogenetic signal in all data subsets as a precondition for the framework showing that habitat conditions diverge linearly with phylogenetic distance. We found a phylogenetic signal on fractions of 0.1–0.25 of maximum phylogenetic distances beyond which a significant correlation was lost (Supplementary Fig. S6). Similarly, we tested whether a phylogenetic signal was also present on organism level by relating habitat preferences of ASVs to phylogenetic distances. It showed a linear increase of habitat differences and phylogenetic distance between pairs of ASVs until a phylogenetic distance of 12% for prokaryotic and 24% for eukaryotic ASVs (Supplementary Fig. S7). The outcome of this analysis shows that homogeneous selection was the dominant mechanism in the prokaryotic 0.2–3 µm fraction at all depths with the highest proportions explained in the upper 60 m, 55–60% (Fig. 5A). In the prokaryotic 3–8 µm and >8 µm fractions, homogeneous selection was also the dominant mechanism in the upper 60 m, explaining 45–65% (Fig. 5A). At these depths, dispersal limitation was the second most important mechanism for the prokaryotic communities of all size fractions but with almost similar proportions as homogeneous selection at 100 m depth. In the mesopelagic, drift was most important for the 3–8 µm and >8 µm fractions (Fig. 5A). For eukaryotic communities homogeneous selection was also the relatively most important mechanism at 20 and 60 m depth (55% and 60%) whereas at all other depths drift was the most important mechanism contributing >50% (Fig. 5A). Dispersal limitation was always of minor importance for eukaryotic community assembly explaining <15% (Fig. 5A).

As temperature differed greatly along the transect together with the changing composition of the prokaryotic communities in the three size fractions we examined the partitioning of the different ecological mechanisms with increasing temperature difference. The results show that homogeneous selection was the dominant mechanism in the 0.2–3 µm size fraction at temperature differences between 4 and 16 °C in the epi- as well as mesopelagic. At greater temperature differences, the relative proportion of homogeneous selection continuously decreased whereas that of dispersal limitation increased and became the most important mechanism above a difference of 16 °C (Supplementary Fig. S8). This was also true for the prokaryotic 3–8 µm and >8 µm fractions in the epipelagic. Drift was most important at low-temperature difference in all three prokaryotic size fractions but relatively most important in the 3–8 µm and >8 µm fractions, in agreement with the results above. The eukaryotic communities were mainly affected by drift and homogenous selection, with decreasing importance of the latter at increasing temperature differences in the epipelagic. Interestingly, heterogeneous selection, generally of minor importance, was most important in the prokaryotic 3–8 µm and >8 µm fractions and especially in the eukaryotic >8 µm fraction in the mesopelagic at the highest temperature differences. The latter results, however, are based on relatively few data as only few samples were available with this high-temperature difference.

We further analyzed how environmental and biotic variables individually affect the structure of prokaryotic and eukaryotic communities and thus β-diversity along the transect. Therefore, we used published data of 21 variables assessed at all stations and depths during the same cruises that generated the amplicon data [30] and analyzed by PERMANOVA the proportion of each variable alone affecting community variance along the transect assessed by the β-diversity indices Bray–Curtis, UniFrac, TINA and PINA. In all 72 analyses, except in six mesopelagic data subsets, the TINA index explained the highest percentage of community variance. Province, temperature and leucine aminopeptidase, a measure of prokaryotic protein/polypeptide break-down, explained in all three epipelagic size fractions >70% of the variance and chlorophyll *a*, particulate organic nitrogen, oxygen, dissolved NO_x_, salinity and prokaryotic cell numbers also 50–65% (Fig. 5B). The three other indices explained much less of the variance and the Bray–Curtis index in most cases least. In the mesopelagic, explained variance of most variables was lower than in the epipelagic (Supplementary Fig. S9).

## Discussion

Our results show that distance decay patterns of the similarity of microbial communities between subantarctic and subarctic regions of the Pacific Ocean assessed by three independent approaches are unimodal and rather similar with the highest dissimilarity in intermediate distance ranges and lowest dissimilarities at shortest and longest distances. Bray–Curtis distance decay patterns show the net-effect of species turnover with geographic distance. The UniFrac analysis, considering phylogenetic signals, here on the level of taxonomic families, yielded also highest dissimilarities at intermediate distances for two phylogenetic clusters, clusters 1 and 2. This indicates that selection is an important mechanism for species turnover, in particular in the two clusters. These clusters include, but are not limited to, the most abundant families [30] (Fig. 2A). They consist of different numbers of genera, species and ASVs and selection may lead to diversification on the genus or species level along an environmental gradient and most pronounced in the intermediate distance range. The distinct distance decay patterns of each of the four clusters indicate, though, that selection acts differently upon the families of each cluster, presumably by the different effects of environmental and biotic variables along the transect. On the other hand, the high phylogenetic similarity among samples from short and large distances in clusters 1 and 2 also indicates the large impact of homogeneous selection, and relatively lower importance of dispersal and drift on species turnover. The increasing importance of dispersal limitation and decreasing importance of homogeneous selection with increasing temperature difference (Supplementary Fig. S8) indicates that homogenous selection was the most important ecological mechanism for the assembly of prokaryotic communities in regions not too different regarding temperature. In this context it is important to consider that selection as an ecological mechanism addresses the composition of the microbial community by different phylogenetic lineages and not the selection pressure on populations on the genus and (sub)species level. Hence, homogeneous selection leads to communities with a more similar composition along a geographic or temperature gradient and heterogeneous selection to communities with a less similar composition [15]. Our analysis of a higher relative importance of homogeneous selection at low-temperature differences and a relatively higher importance of dispersal limitation, and reduced importance of homogeneous selection, at higher temperature difference is consistent with this notion. It reflects the adaptation of prokaryotic communities as a whole to the given environmental conditions. The role and significance of selection on populations on the (sub)species level for the SAR11 clade I is discussed further below.

Bipolar distribution patterns of oceanic microbial communities have been reported previously [8, 11, 51]. Our results confirm these patterns but specify them by disentangling the relative significance of different ecological mechanisms and considering taxon interaction structure causing species sorting and community assembly. Each approach we applied exhibits distinct features regarding dissimilarity values. The TINA index explained the largest percentage of the variations of environmental and in particular biotic variables affecting β-diversity of the prokaryotic and eukaryotic communities along the transect but a consistently higher percentage of the former. These findings indicate the importance of biotic and taxon-associated interactions among the different members in particular of the prokaryotic communities. Even though the TINA index does not address specific interactions among microorganisms, it emphasizes the significance of such interactions for community assembly and establishing biogeographic patterns [18]. Interactions among prokaryotic and eukaryotic community members have been shown to be of pivotal significance in particular under nutrient-limiting conditions such as in pelagic marine systems where genome-streamlined prokaryotes constitute microbial communities to a great extent [52], complemented by microeukaryotes and tightening the mutual exchange of growth factors such as B vitamins [53]. The Black Queen hypothesis captures the importance of species interactions at such conditions, as genome-streamlined prokaryotes are assumed to discard the genetic potential for the biosynthesis of public goods available from other pro- and eukaryotic community members [54]. These interactions include the supply and use of vitamin B_1_ and its building blocks, B_7_ [55, 56] and B_12_ [53, 57–59] by auxotrophic and prototrophic prokaryotes and eukaryotes but also include sharing polymer breakdown products [60]. There are presumably many more so far unrecognized positive interactions among prokaryotic community members regarding metabolite cross-feeding and also between prokaryotes and phytoplankton algae [61–63], which further tighten these interactions.

The power of the TINA index and thus of taxon interactions relative to other indices not considering such interaction structures for assessing β-diversity of microbial communities has been shown for marine microplankton communities of a subset of tropical and subtropical stations of the Tara Ocean data set [18]. There is only one other study employing the TINA index for prokaryotic and picoeukaryotic oceanic communities, but restricted to epipelagic tropical and subtropical regions of the global oceans sampled during the Malaspina expedition. This study also found that this index accounted for a much higher percentage of variance than other indices of the biogeographic variability of prokaryotic community composition as a function of water temperature but not for provincialism [24]. The TINA index in this study represented only 50% and 25% of the prokaryotic community variance for each variable. For picoeukaryotes, however, no difference was detected regarding temperature and for provincialism the TINA index did not yield significant results. The percentages of this index for prokaryotic communities we found for our basin-wide data set of the Pacific Ocean are for most environmental and biotic variables greater than 60% and reach 90% for temperature and province and for microeukaryotes we also found significant results and percentages of 25 to 50%. These findings emphasize the significance of these variables for the interactions and assembly of prokaryotic as well as eukaryotic oceanic microbial communities. In the view of these interactions, it does not seem surprising that the distance decay dissimilarities of the TINA index are lower than those of the other indices based just on compositional or phylogenetic distance between individual taxa. Community interactions are a stable phenomenon irrespective of geographic distance, temperature gradients or biogeographic regions, e.g. due to the production of and dependence on public goods of distinct community members. This implies that interaction-adjusted indices will produce generally lower dissimilarities as they quantify the overlap of groups of co-occurring organisms, not only the presence of different taxa, which may be interchanged by other community members due to functional redundancy.

### Significance of ecological mechanisms for community assembly

**Fig. 6:**
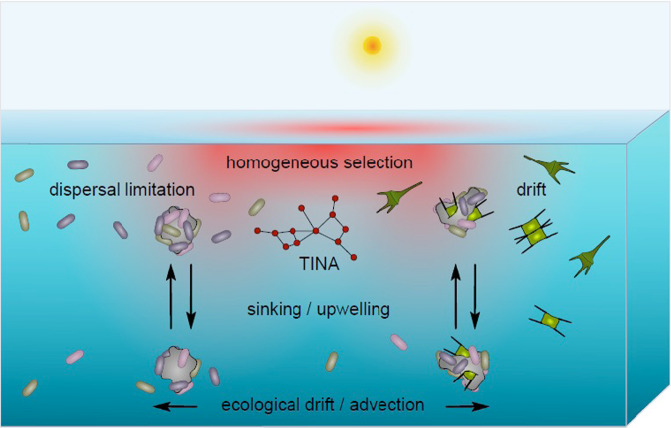
Conceptual framework of how ecological mechanisms and TINA index affect microbial pelagic communities. Displayed is how the ecological mechanisms selection, dispersal and drift and organism interactions by the TINA index affect the assembly and structuring of prokaryotic and eukaryotic microbial communities in epi- and mesopelagic waters in an ocean basin.

The analytical framework [17] we applied to disentangle the relative significance of selection, dispersal and drift for the assembly of microbial communities provides the most comprehensive approach, despite some limitations and assumptions [19], but has never been applied to the microbiome of an ocean basin. Our results demonstrate that homogeneous selection is of relatively greatest significance for the assembly of prokaryotic communities in the epipelagic Pacific whereas for that of microeukaryotic communities drift is of relatively greater importance, as it is for PA prokaryotic communities in the upper mesopelagic. However, it must be kept in mind that all three ecological mechanisms act together, but differently, to shape biogeographic patterns of the prokaryotic and eukaryotic microbial communities in the epi- and upper mesopelagic Pacific. The epipelagic encompasses different well-separated water masses along the transect [12, 26, 30]. These water masses, such as the subtropical gyres, the equatorial upwelling and the polar frontal region, differ greatly in their extension. Their environmental constraints obviously make homogeneous selection the dominant factor for shaping prokaryotic communities. As discussed earlier the greatest similarity of the communities in regions of lowest and highest distance, here in particular of lowest distance, reflects the formation of microbial communities well adapted to the given environmental and biotic conditions in one or closely adjacent regions with rather similar environmental conditions. Dispersal limitation also plays an important role as shaping factor, obviously reflecting the separation of the different water masses but also limited mixing within the large gyres. The different relative partitioning of homogeneous selection and dispersal limitation with increasing temperature difference reflects well the interplay of both mechanisms at different hydrographic conditions (Supplementary Fig. S8). In other studies dispersal limitation was shown to be an important mechanism for the assembly of prokaryotic communities, such as in the Southern Ocean [21] or in a coast-to-open sea gradient with increasing significance of this mechanism from the surface to the sea floor [13]. The only other study which assessed the relative significance of the three mechanisms for shaping prokaryotic and also picoeukaryotic communities in oceanic systems encompasses the global subtropical and tropical epipelagic oceans together and is based on samples collected during the Malaspina expedition [24]. For prokaryotic communities this study found that drift contributed 45% of the total whereas homogeneous selection and dispersal limitation each only about 26%. For picoeukaryotic communities dispersal limitation explained about 65%. The differences to our results are presumably due to the inclusion of water masses of different oceans and the restriction to subtropical and tropical regions. In fact, a follow-up study of these Malaspina data showed that the similarity of prokaryotic and picoeukaryotic communities within samples of the Atlantic, Indian and Pacific Ocean was higher than among these oceans [64]. In our study, drift was the dominant factor in the mesopelagic for PA prokaryotic communities whereas homogeneous selection was also the most important factor for the free-living prokaryotic communities in this layer. The import of PA prokaryotes from the epipelagic by sinking particles during regional phytoplankton bloom events [28] and advection of mesopelagic water masses, e.g. the Pacific Equatorial and the Antarctic and Pacific Intermediate Waters, presumably leads to stochastic advection and circulation of PA prokaryotes into mesopelagic depths (Fig. 6). Advective currents are particularly pronounced in the equatorial region of the central Pacific [65]. The much smaller population size of the PA relative to the free-living prokaryotic community in the 0.2–3 µm size fraction, not exceeding 5–10% in the epi- and mesopelagic Pacific [30, 66], presumably contributes to the significance of drift [14, 19]. Additionally, population dynamics of PA prokaryotic communities are greater than of free-living communities by the active colonization and degradation of particles by prokaryotes, further contributing to drift effects. The more even distribution of prokaryotic communities over longer distances, as found in the upper mesopelagic Pacific (Fig. 4A, C) appears to be even more pronounced in the bathypelagic of the Pacific and other ocean basins. Studies of bathypelagic free-living prokaryotic communities in the global oceans reported only little differences within basins but distinct differences among ocean basins [64, 67].

It was surprising that drift was the dominant factor in our study for shaping eukaryotic communities except at two epipelagic depths. This stochastic factor has rarely been shown to be important and almost never the dominant factor for community assembly of microorganisms [14, 17, 19] except for prokaryotic communities in the mentioned study of the global subtropical and tropical oceans [24] (see above). The dominant role of homogeneous selection at 20 m and 60 m depth for the assembly of microeukaryotic communities is consistent with that for prokaryotic communities at these depths. At 100 m, the lower margin of the epipelagic, and in the upper mesopelagic, aggregation of eukaryotic microorganisms, sinking, decomposition and regional irregular upwelling of mesoscale eddies [25], injecting nutrients into epipelagic waters and bringing eukaryotes into more favourable growth conditions, may explain the great significance of this stochastic factor for shaping eukaryotic microbial communities at these depths in the Pacific Ocean (Fig. 6). The low population size and the relatively low number of eukaryotic ASV reads in our data set may have further enhanced the effect of drift. On the other hand, our results and those of Logares et al. [24] indicate that drift is an important mechanism for the spreading of eukaryotes in the oceans, and also for PA prokaryotes in the mesopelagic and presumably in the bathypelagic as well [67].

### Diversification of the SAR11 Clade 1 along the transect

The important role of selection, not as an ecological factor but for niche separation of populations on the subspecies level, equivalent to the ecological mechanism heterogeneous selection, is reflected by the UniFrac analysis of the SAR11 Clade I ecotype populations with a continuous divergence from equatorial to subpolar waters in both hemispheres along the transect. This analysis is consistent with results of a recent study of the global pangenome of the SAR11 Clade I breaking down this clade into several subclades restricted to distinct biogeographic regions from tropical to polar waters [68]. These authors identified environmentally mediated selection as the major driver of diversification, in line with our analyses showing that this diversification occurs also in the SAR11 Clade I in the Pacific Ocean between subantarctic and subarctic waters. Biogeographic patterns based on gene variants of ecotypes of major taxa of prokaryotic communities in near-surface waters between the Southern Ocean and the temperate North Atlantic further indicate the significance of heterogeneous selection on the (sub)species level and dispersal limitation in ocean basins [22].

### Concluding remarks

Our study disentangled the relative significance of the three ecological factors and the TINA index for shaping latitudinal biogeographic patterns of free-living and PA prokaryotic and eukaryotic microbial communities in the epi- and mesopelagic Pacific and their hydrographic features (Fig. 6). The detailed analyses are based on a data set spanning from subantarctic to subarctic regions of the epi- and upper mesopelagic Pacific Ocean and covering its major biogeographic provinces. As this is the largest ocean, occupying about 50% of the global oceans, we assume that our findings are valid also for other basin-scale oceanic systems such as the Atlantic and Indian Ocean. Each ocean, however, may also exhibit distinct features because of its specific structure and circulation patterns as shown for latitudinal gradients of eukaryotic micro- and macroorganisms [69] and prokaryotic communities in the  epi- and bathypelagic [64, 67]. Our findings provide a framework to compare oceanic systems and to forecast how ecological mechanisms and taxon co-occurrence affect species sorting and community assembly under changing climatic and hydrographic conditions and circulation patterns in the global ocean.

## Supplementary information

Supplemental Tables and Figures

## Data Availability

The scripts for the bioinformatic pipeline are available at https://github.com/jcmcnch/eASV-pipeline-for-515Y-926R. Bioinformatic processing of sequence data was executed within a standardized CONDA environment to improve reproducibility of results. Environmental and biotic variables used for the explanation of community variation are available at https://doi.pangaea.de/10.1594/PANGAEA.918500. Amplicon sequence data is deposited in the European Nucleotide Archive (ENA) under project accession number PRJEB51015.

## References

[1] Nelson G (1978). From Candolle to croizat: comments on the history of biogeography. J Hist Biol.

[2] Lomolino MV, Riddle BR, Whittaker RJ, Brown JH. Biogeography. Sunderland, MA: Sinauer Associates; 2005. p. 752

[3] Wang J, Soininen J, Zhang Y, Wang B, Yang X, Shen J (2011). Contrasting patterns in elevational diversity between microorganisms and macroorganisms. J Biogeogr.

[4] Treseder KK, Maltz MR, Hawkins BA, Fierer N, Stajich JE, Mcguire KL (2014). Evolutionary histories of soil fungi are reflected in their large-scale biogeography. Ecol Lett.

[5] Meyer KM, Memiaghe H, Korte L, Kenfack D, Alonso A, Bohannan BJM (2018). Why do microbes exhibit weak biogeographic patterns?. ISME J.

[6] Lindström ES, Langenheder S (2012). Local and regional factors influencing bacterial community assembly. Environ Microbiol Rep.

[7] Ghiglione JF, Galand PE, Pommier T, Pedrós-Alió C, Maas EW, Bakker K (2012). Pole-to-pole biogeography of surface and deep marine bacterial communities. Proc Natl Acad Sci USA.

[8] Sul WJ, Oliver TA, Ducklow HW, Amaral-Zettlera LA, Sogin ML (2013). Marine bacteria exhibit a bipolar distribution. Proc Natl Acad Sci USA.

[9] Sunagawa S, Coelho LP, Chaffron S, Kultima JR, Labadie K, Salazar G (2015). Structure and function of the global ocean microbiome. Science.

[10] de Vargas C, Audic S, Henry N, Decelle J, Mahé F, Logares R (2015). Eukaryotic plankton diversity in the sunlit ocean. Science.

[11] Milici M, Tomasch J, Wos-Oxley ML, Decelle J, Jáuregui R, Wang H (2016). Bacterioplankton biogeography of the Atlantic ocean: a case study of the distance-decay relationship. Front Microbiol.

[12] Raes EJ, Bodrossy L, Van De Kamp J, Bissett A, Ostrowski M, Brown MV (2018). Oceanographic boundaries constrain microbial diversity gradients in the south pacific ocean. Proc Natl Acad Sci USA.

[13] Wu W, Lu HP, Sastri A, Yeh YC, Gong GC, Chou WC (2018). Contrasting the relative importance of species sorting and dispersal limitation in shaping marine bacterial versus protist communities. ISME J.

[14] Vellend M (2010). Conceptual synthesis in community ecology. Q Rev Biol.

[15] Hanson CA, Fuhrman JA, Horner-Devine MC, Martiny JBH (2012). Beyond biogeographic patterns: Processes shaping the microbial landscape. Nat Rev Microbiol.

[16] Nemergut DR, Schmidt SK, Fukami T, O’Neill SP, Bilinski TM, Stanish LF (2013). Patterns and processes of microbial community assembly. Microbiol Mol Biol Rev.

[17] Stegen JC, Lin X, Fredrickson JK, Chen X, Kennedy DW, Murray CJ (2013). Quantifying community assembly processes and identifying features that impose them. ISME J.

[18] Schmidt TSB, Matias Rodrigues JF, Von Mering C (2017). A family of interaction-adjusted indices of community similarity. ISME J.

[19] Zhou J, Ning D (2017). Stochastic community assembly: does it matter in microbial ecology?. Microbiol Mol Biol Rev.

[20] Djurhuus A, Port J, Closek CJ, Yamahara KM, Romero-maraccini O, Walz KR (2017). Evaluation of filtration and DNA extraction methods for environmental DNA biodiversity assessments across multiple trophic levels. Front Mar Sci.

[21] Wang ZB, Sun YY, Li Y, Chen XL, Wang P, Ding HT (2020). Significant bacterial distance-decay relationship in continuous, well-connected southern ocean surface water. Micro Ecol.

[22] Dlugosch L, Pohlein A, Wemheuer B, Pfeiffer B, Badewien T, Daniel R (2022). Significance of gene variants for the functional biogeography of the near-surface Atlantic Ocean microbiome. Nat Commun.

[23] Lozupone C, Knight R (2005). UniFrac: A new phylogenetic method for comparing microbial communities. Appl Environ Microbiol.

[24] Logares R, Deutschmann IM, Junger PC, Giner CR, Krabberød AK, Schmidt TSB (2020). Disentangling the mechanisms shaping the surface ocean microbiota. Microbiome.

[25] Doblin MA, Petrou K, Sinutok S, Seymour JR, Messer LF, Brown MV (2016). Nutrient uplift in a cyclonic eddy increases diversity, primary productivity and iron demand of microbial communities relative to a western boundary current. PeerJ.

[26] Polovina JJ, Howell E, Kobayashi DR, Seki MP (2001). The transition zone chlorophyll front, a dynamic global feature defining migration and forage habitat for marine resources. Prog Oceanogr.

[27] Karl DM, Church MJ (2017). Ecosystem structure and dynamics in the north pacific subtropical gyre: new views of an old ocean. Ecosystems.

[28] Mestre M, Ruiz-González C, Logares R, Duarte CM, Gasol JM, Sala MM (2018). Sinking particles promote vertical connectivity in the ocean microbiome. Proc Natl Acad Sci USA.

[29] Balmonte JP, Simon M, Giebel HA, Arnosti C (2021). A sea change in microbial enzymes: Heterogeneous latitudinal and depth-related gradients in bulk water and particle-associated enzymatic activities from 30°S to 59°N in the Pacific Ocean. Limnol Oceanogr.

[30] Giebel H-A, Arnosti C, Badewien TH, Bakenhus I, Balmonte JP, Billerbeck S (2021). Microbial growth and organic matter cycling in the Pacific Ocean along a latitudinal transect between subarctic and subantarctic waters. Front Mar Sci.

[31] Milici M, Tomasch J, Wos-Oxley ML, Wang H, Jáuregui R, Camarinha-Silva A (2016). Low diversity of planktonic bacteria in the tropical ocean. Sci Rep.

[32] Longhurst AR. Ecological geography of the sea. San Diego, USA: Academic Press; 2007.

[33] Parada AE, Needham DM, Fuhrman JA (2016). Every base matters: Assessing small subunit rRNA primers for marine microbiomes with mock communities, time series and global field samples. Environ Microbiol.

[34] Milke F, Sanchez-Garcia S, Dlugosch L, McNichol J, Fuhrman J, Simon M (2022). Composition and biogeography of pro- and eukaryotic communities in the Atlantic Ocean: primer choice matters. Front Microbiol.

[35] Vaulot D, Geisen S, Mahé F, Bass D (2022). pr2-primers: An 18S rRNA primer database for protists. Mol Ecol Resour.

[36] Yeh YC, McNichol J, Needham DM, Fichot EB, Berdjeb L, Fuhrman JA (2021). Comprehensive single-PCR 16S and 18S rRNA community analysis validated with mock communities, and estimation of sequencing bias against 18S. Environ Microbiol.

[37] Quast C, Pruesse E, Yilmaz P, Gerken J, Schweer T, Yarza P (2013). The SILVA ribosomal RNA gene database project: Improved data processing and web-based tools. Nucleic Acids Res.

[38] Guillou L, Bachar D, Audic S, Bass D, Berney C, Bittner L (2013). The Protist Ribosomal Reference database (PR2): a catalog of unicellular eukaryote Small Sub-Unit rRNA sequences with curated taxonomy. Nucleic Acids Res.

[39] Bolyen E, Rideout JR, Dillon MR, Bokulich NA, Abnet CC, Al-Ghalith GA (2019). Reproducible, interactive, scalable and extensible microbiome data science using QIIME 2 (Nature Biotechnology, (2019), 37, 8, (852-857), 10.1038/s41587-019-0209-9). Nat Biotechnol.

[40] Callahan BJ, McMurdie PJ, Rosen MJ, Han AW, Johnson AJA, Holmes SP (2016). DADA2: High-resolution sample inference from Illumina amplicon data. Nat Methods.

[41] Friedman J, Alm EJ (2012). Inferring correlation networks from genomic survey data. PLoS Comput Biol.

[42] Bodenhofer U, Bonatesta E, Horejš-Kainrath C, Hochreiter S (2015). Msa: an R package for multiple sequence alignment. Bioinformatics.

[43] Kaufman L, Rousseeuw PJ. Finding groups in data: an introduction to cluster analysis. Hoboken NJ, USA: John Wiley & Sons; 2009.

[44] Pruesse E, Peplies J, Glöckner FO (2012). SINA: Accurate high-throughput multiple sequence alignment of ribosomal RNA genes. Bioinformatics.

[45] Price MN, Dehal PS, Arkin AP (2010). FastTree 2 - approximately maximum-likelihood trees for large alignments. PLoS ONE.

[46] Losos JB (2008). Phylogenetic niche conservatism, phylogenetic signal and the relationship between phylogenetic relatedness and ecological similarity among species. Ecol Lett.

[47] Stegen JC, Lin X, Konopka AE, Fredrickson JK (2012). Stochastic and deterministic assembly processes in subsurface microbial communities. ISME J.

[48] Fine PVA, Kembel SW (2011). Phylogenetic community structure and phylogenetic turnover across space and edaphic gradients in western Amazonian tree communities. Ecography.

[49] Chase JM, Kraft NJB, Smith KG, Vellend M, Inouye BD (2011). Using null models to disentangle variation in community dissimilarity from variation in α-diversity. Ecosphere.

[50] NASA Goddard Space Flight Center, Ocean Ecology Laboratory OBPG. Moderate-resolution Imaging Spectroradiometer (MODIS) aqua chlorophyll data. https://oceancolor.gsfc.nasa.gov/data/10.5067/AQUA/MODIS/L3B/CHL/2018/. Accessed 13 Nov 2020.

[51] Pommier T, Douzery EJP, Mouillot D (2012). Environment drives high phylogenetic turnover among oceanic bacterial communities. Biol Lett.

[52] Giovannoni SJ, Cameron Thrash J, Temperton B (2014). Implications of streamlining theory for microbial ecology. ISME J.

[53] Sañudo-Wilhelmy SA, Gómez-Consarnau L, Suffridge C, Webb EA (2014). The role of B vitamins in marine biogeochemistry. Ann Rev Mar Sci.

[54] Morris JJ, Lenski RE, Zinser ER (2012). The black queen hypothesis: evolution of dependencies through adaptive gene loss. MBio.

[55] Carini P, Campbell EO, Morré J, Sañudo-Wilhelmy SA, Cameron Thrash J, Bennett SE (2014). Discovery of a SAR11 growth requirement for thiamin’s pyrimidine precursor and its distribution in the Sargasso Sea. ISME J.

[56] Wienhausen G, Bruns S, Sultana S, Dlugosch L, Groon L, Wilkes H, et al. The overlooked role of a biotin precursor for marine bacteria - desthiobiotin as an escape route for biotin auxotrophy. ISME J. 2022. 10.1038/s41396-022-01304-w. 10.1038/s41396-022-01304-w PMC9561691 35963899

[57] Biller SJ, Coe A, Chisholm SW (2016). Torn apart and reunited: Impact of a heterotroph on the transcriptome of Prochlorococcus. ISME J.

[58] Sokolovskaya OM, Shelton AN, Taga ME (2020). Sharing vitamins: cobamides unveil microbial interactions. Science.

[59] Wienhausen G, Dlugosch L, Jarling R, Wilkes H, Giebel H-A, Simon M (2022). Availability of vitamin B12 and its lower ligand intermediate a-ribazole impact prokaryotic and protist communities in oceanic systems. ISME J.

[60] Reintjes G, Arnosti C, Fuchs B, Amann R (2019). Selfish, sharing and scavenging bacteria in the Atlantic Ocean: a biogeographical study of bacterial substrate utilisation. ISME J.

[61] Bertrand EM, McCrow JP, Moustafa A, Zheng H, McQuaid JB, Delmont TO (2015). Phytoplankton-bacterial interactions mediate micronutrient colimitation at the coastal Antarctic sea ice edge. Proc Natl Acad Sci USA.

[62] Amin SA, Hmelo LR, Van Tol HM, Durham BP, Carlson LT, Heal KR (2015). Interaction and signalling between a cosmopolitan phytoplankton and associated bacteria. Nature.

[63] Shibl AA, Isaac A, Ochsenkühn MA, Cárdenas A, Fei C, Behringer G (2020). Diatom modulation of select bacteria through use of two unique secondary metabolites. Proc Natl Acad Sci USA.

[64] Villarino E, Watson JR, Chust G, Woodill AJ, Klempay B, Jonsson B (2022). Global beta diversity patterns of microbial communities in the surface and deep ocean. Glob Ecol Biogeogr.

[65] Cravatte S, Kestenare E, Marin F, Dutrieux P, Firing E (2017). Subthermocline and intermediate zonal currents in the tropical Pacific Ocean: Paths and vertical structure. J Phys Oceanogr.

[66] Cho BC, Azam F (1988). Major role of bacteria in biogeochemical fluxes in the ocean’s interior. Nature.

[67] Salazar G, Cornejo-Castillo FM, Benítez-Barrios V, Fraile-Nuez E, Álvarez-Salgado XA, Duarte CM (2016). Global diversity and biogeography of deep-sea pelagic prokaryotes. ISME J.

[68] Delmont TO, Kiefl E, Kilinc O, Esen OC, Uysal I, Rappé MS (2019). Single-amino acid variants reveal evolutionary processes that shape the biogeography of a global SAR11 subclade. Elife.

[69] Hillebrand H (2004). On the generallity of the latutinal diversity gradient. Am Nat.

