# Supplementary material for: Selection, drift and community interactions shape microbial biogeographic patterns in the Pacific Ocean
Source: ISME J. 2022 Sep 17;16(12):2653–65. doi: 10.1038/s41396-022-01318-4 (PMC9666467; doi:10.1038/s41396-022-01318-4)
Supplement: Supplementary file 1 — Supplemental Tables and Figures [file 41396_2022_1318_MOESM1_ESM.pdf]

The ISME Journal, provisionally accepted

Selection, drift and community interactions shape microbial biogeographic patterns in the Pacific Ocean

Felix Milke[1], Irene Wagner-Doebler[2], Gerrit Wienhausen[1], Meinhard Simon[1,3 *]

[1] Institute for Chemistry and Biology of the Marine Environment, University of Oldenburg, Carl von Ossietzky Str. 9-11, D-26129 Oldenburg, Germany

[2] Institute of Microbiology, Technical University of Braunschweig, D-38106 Braunschweig, Germany

[3] Helmholtz Institute for Functional Marine Biodiversity at the University of Oldenburg (HIFMB), Ammerländer Heerstraße 231, D-26129 Oldenburg, Germany

* Corresponding author (m.simon@icbm.de)

Supplementary Material

Table S1
Sequencing statistics

Table S2
Clusters 1 to 4 of the UniFrac analyses

Figure S1
Contour graphs of chlorophyll fluorescence and salinity in the epi- and mesopelagic between subantarctic and subarctic waters of the Pacific Ocean

Figure S2
Composition of eukaryotic communities in the biogeographic provinces of the epi- and mesopelagic Pacific between subantarctic and subarctic regions.

Figure S3
Distribution patterns of prokaryotic phylogenetic clusters 1 to 4 in the FL, SPA and LPA size fractions along the transect between subantarctic and subarctic waters in the epi- and mesopelagic Pacific.

Figure S4
TINA dissimilarity as a function of temperature differences of stations and depths of FL, SPA and LPA epi- and mesopelagic prokaryotic communities between the subantarctic and subarctic Pacific.

Figure S5
Heatmap of abundances of SAR11 Clade I-ASVs in the epipelagic along the transect.

Figure S6
Mantel-correlogram that shows linear correlation between bins of phylogenetic dissimilarity and habitat differences for prokaryotic communities of each size-fraction and eukaryotic communities.

Figure S7
Habitat differences between pairs of ASVs against their phylogenetic dissimilarity for FL, SPA and LPA prokaryotic communities.

Figure S8
Relative importance of ecological mechanisms with increasing difference of water temperature.

Figure S9
Community variance individually explained by single environmental variables for different dissimilarity indices (Bray-Curtis, UniFrac, TINA and PINA) for FL, SPA and LPA prokaryotic communities in mesopelagic depths.

Table S1

Number of ASV reads recruited in the 0.2-3 µm, 3-8 µm and >8 µm size fractions affiliated to *Bacteria*, *Archaea* and eukaryotic microbes amplified by using the V4-V5 primer set of Parada et al., targeting the V4-V5 region of the 16S and 18S rRNA gene fragments.

**Size-Fraction**	**0.22 – 3 µm**	**3 – 8 µm**	**>8 µm**
Bacteria	4,126,515	3,113,797	2,689,158
Archaea	463,066	261,646	243,694
Eukaryotes			1,018,482

Table S2

Members of clusters inferred from patterns between UniFrac dissimilarity and geographic distance along the transect. Clusters identified by hierarchical Silhouette clustering of phylogenetic distance-decay patterns of individual taxonomic families using SILVA132 classification.

**Cluster 1 (n=13)**	**Cluster 2 (n=30)**	**Cluster 3 (n=46)**	**Cluster 4 (n=17)**
BIrii41	Acetobacteraceae	053A03-B-DI-P58	Alcanivoracaceae
SAR11 Clade I	Actinomarinaceae	AB1	Bacteriovoracaceae
SAR11 Clade III	Anaerolineaceae	AEGEAN-169 marine group	Gimesiaceae
SAR 11 Clade IV	Arenicellaceae	Alteromonadaceae	Haliangiaceae
Cyanobiaceae	Bacillaceae	Bdellovibrionaceae	Hyphomonadaceae
Desulfarculaceae	Cellvibrionaceae	Beijerinckiaceae	Litoricolaceae
Ectothiorhodospiraceae	Clade II	Blfdi19	Microscillaceae
Halomonadaceae	Colwelliaceae	Burkholderiaceae	Moraxellaceae
Nisaeaceae	Crocinitomicaceae	Caulobacteraceae	Nocardioidaceae
Parvibaculaceae	Cryomorphaceae	Chitinophagaceae	OCS116 clade
Rubritaleaceae	Enterobacteriaceae	Coxiellaceae	Propionibacteriaceae
Saccharospirillaceae	Flavobacteriaceae	Cyclobacteriaceae	Pseudomonadaceae
Thiotrichaceae	Flavobacteria NS7 marine group	Deinococcaceae	Rhodocyclaceae
	Flavobacteria NS9 marine group	DEV007	Sphingomonadaceae
	Halieaceae	Entomoplasmatales Incertae Sedis	Staphylococcaceae
	Magnetospiraceae	Francisellaceae	Vibrionaceae
	Methylophilaceae	Gemmataceae	Xanthobacteraceae
	Microtrichaceae	Idiomarinaceae	
	Mitochondria	Legionellaceae	
	Nitrincolaceae	Lentisphaeraceae	
	Nitrosopumilaceae	Marinobacteraceae	
	Nitrospinaceae	Methylophagaceae	
	Pirellulaceae	Microbacteriaceae	
	Porticoccaceae	Micrococcaceae	
	Pseudohongiellaceae	Midichloriaceae	
	Rhizobiaceae	Mycobacteriaceae	
	Rhodobacteraceae	Nannocystaceae	
	Rubinisphaeraceae	Nocardiaceae	

	SAR116 clade	Oligoflexaceae	
	Woeseiaceae	P3OB-42	
		Phycisphaeraceae	
		Piscirickettsiaceae	
		Pseudoalteromonadaceae	
		Puniceicoccaceae	
		Rickettsiaceae	
		S25-593	
		Saprospiraceae	
		Shewanellaceae	
		Simkaniaceae	
		Sneathiellaceae	
		Solimonadaceae	
		Spirosomaceae	
		Spongiibacteraceae	
		Thalassospiraceae	
		Thioglobaceae	
		Xanthomonadaceae	

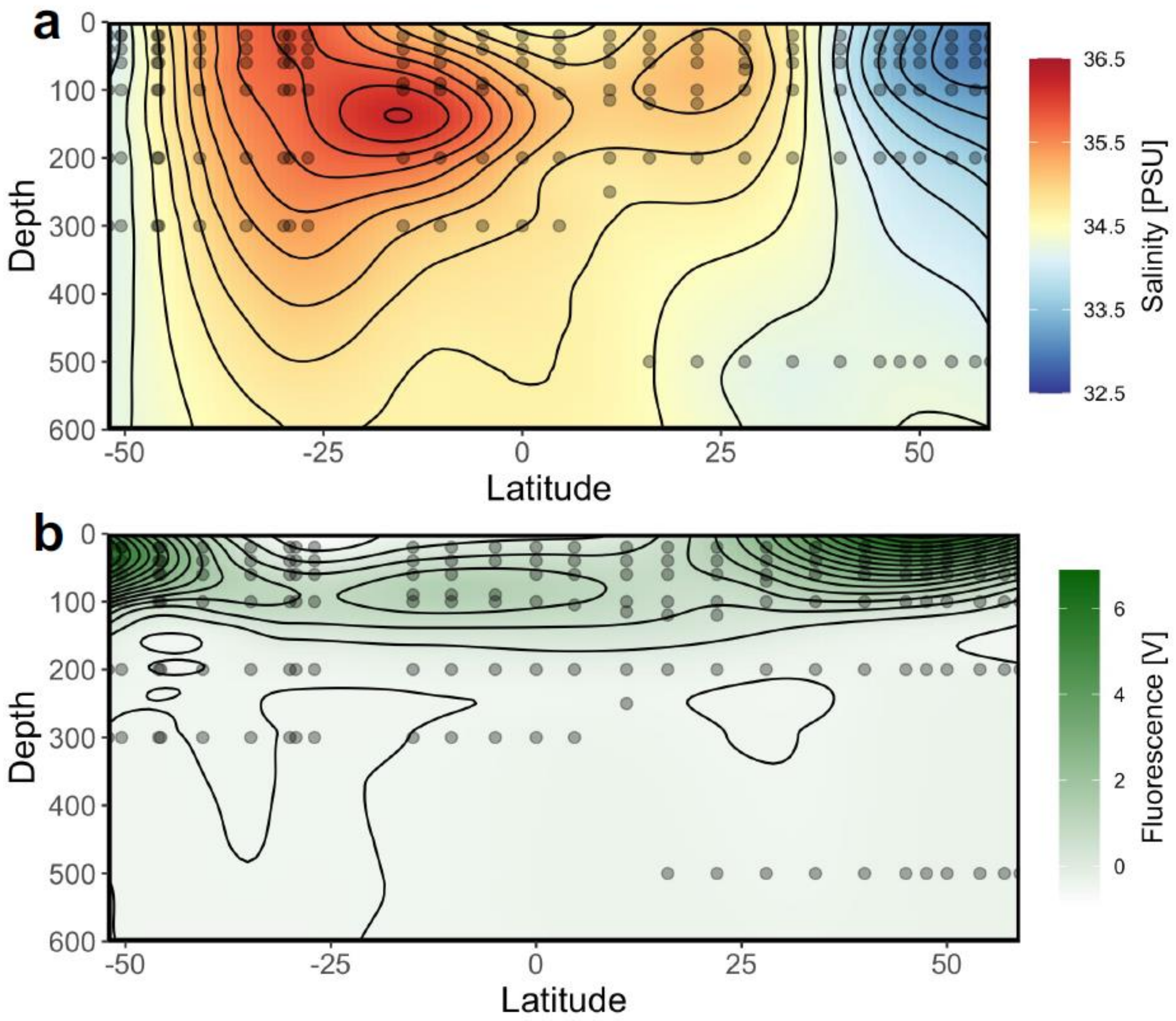

Figure S1. **Contour graphs of chlorophyll fluorescence and salinity in the epi- and mesopelagic between subantarctic and subarctic waters of the Pacific Ocean a**) Contour graph of chlorophyll fluorescence along the transect based on continuous measurements by a fluorescence probe at each station between the surface and 600 m depth. B) Contour graph of salinity along the transect based on continuous measurements by a probe at each station between the surface and 600 m depth. Black dots indicate the depth of sampling.

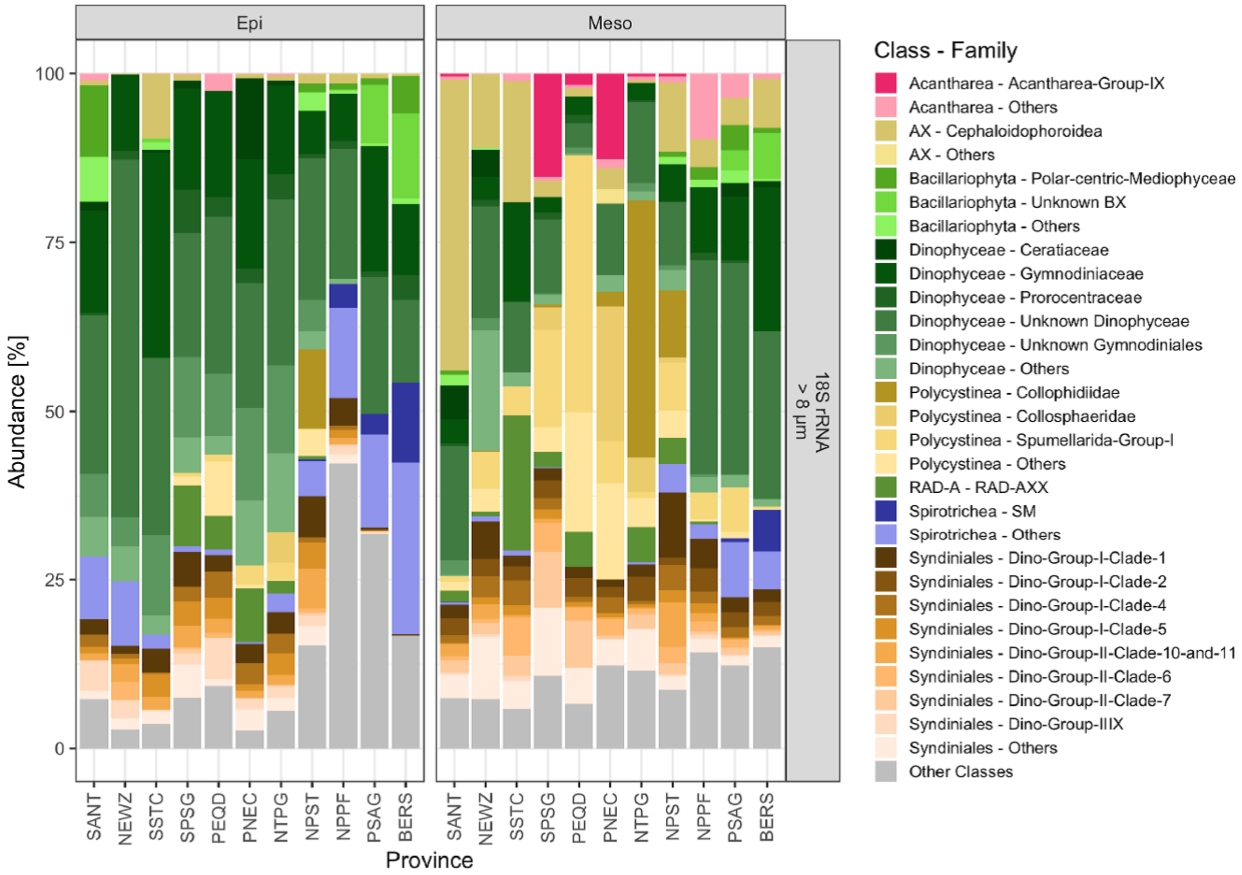

Figure S2: **Composition of eukaryotic communities in the biogeographic provinces of the epi- and mesopelagic Pacific between subantarctic and subarctic regions**. Relative abundance (% of total) of eukaryotic classes and major families in each biogeographic province in the epi- and mesopelagic. Stacked bars of each province are means of all stations and depths of a given province and layer. Other classes include those which constitute <0.8% each.

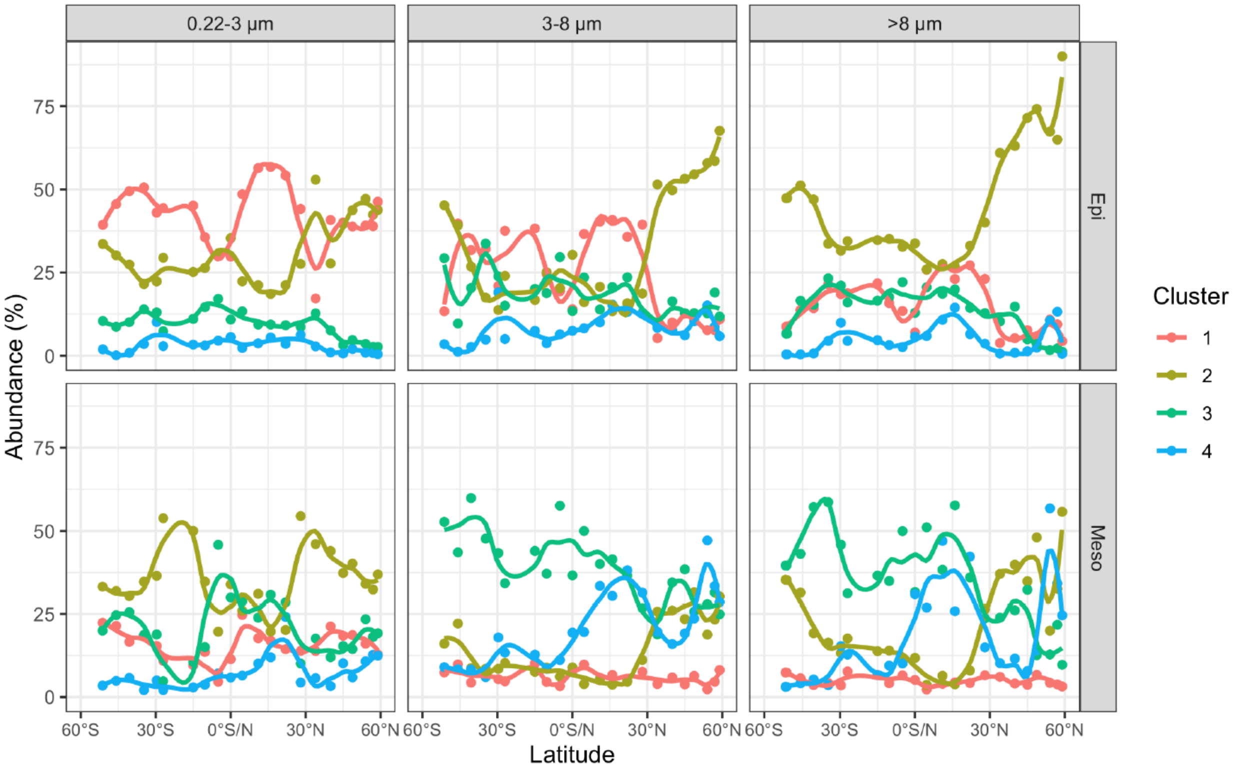

Figure S3 **Distribution patterns of prokaryotic phylogenetic clusters 1 to 4 in the 0.22-3 µm, 3-8 µm and >8 µm size fractions along the transect between subantarctic and subarctic waters in the epi- and mesopelagic Pacific**. Each cluster encompassed phylogenetic families, which are specified in supplementary Table S2. The lines represent loess-fits of the respective cluster abundance along the transect.

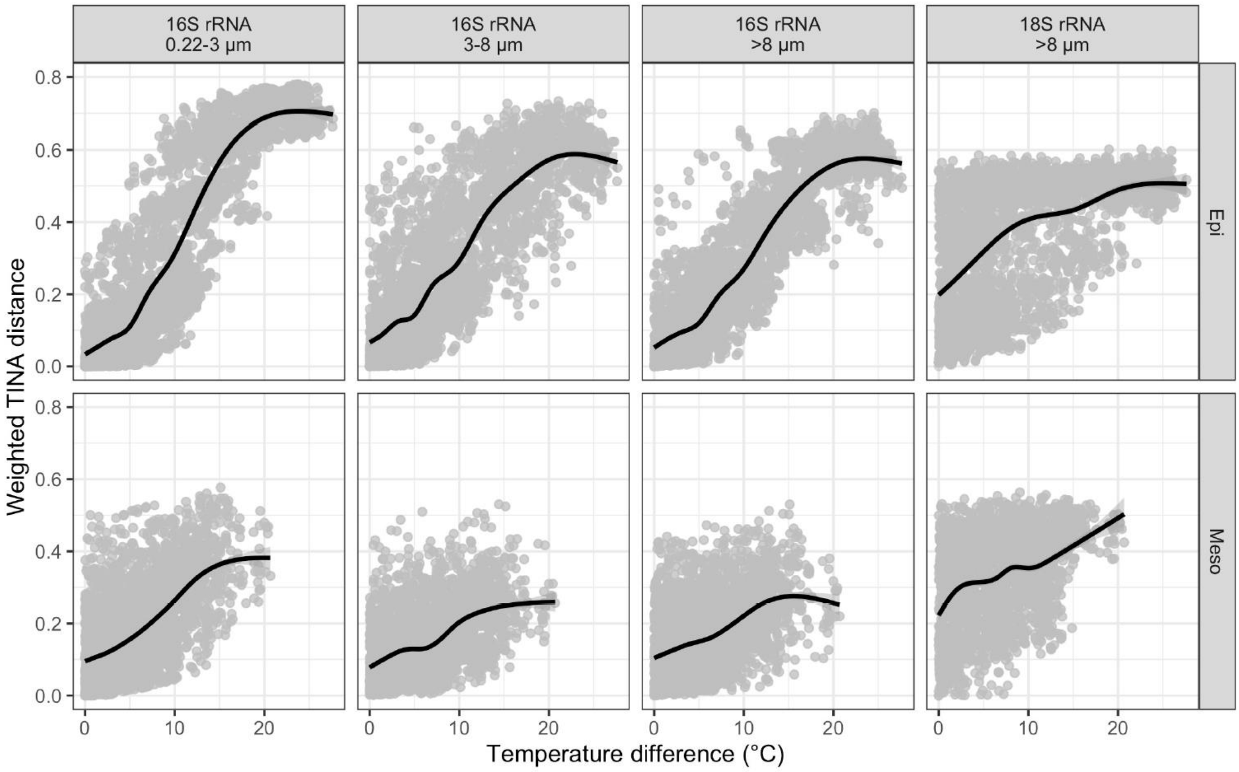

Figure S4 TINA dissimilarity as a function of temperature differences of stations and depths in the 0.22-3 µm, 3-8 µm and >8 µm size fractions of the epi- and mesopelagic prokaryotic (16S rRNA) and eukaryotic (18S rRNA) communities between the subantarctic and subarctic Pacific. Black lines indicate a loess-fit for the respective data subset.

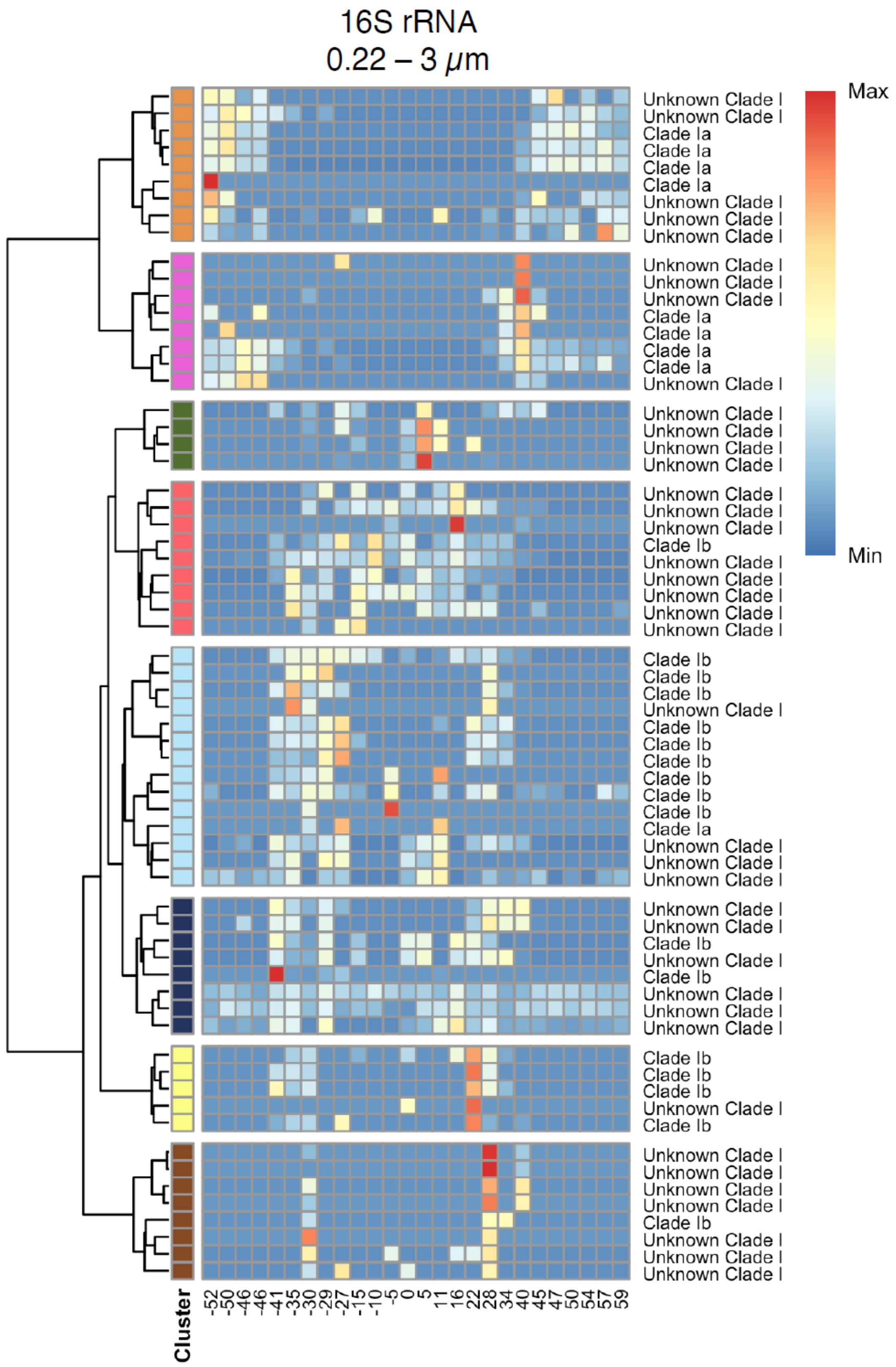

Figure S5 **Heatmap of abundances of SAR11 Clade I-ASVs in the epipelagic along the transect**. Abundance was normalized row-wise and displayed by colour. Genus classification of each ASV based on SILVA132 are shown as names. Rows are sorted by hierarchical clustering based on Bray-Curtis and separated by gaps that represent clusters inferred from silhouette clustering.

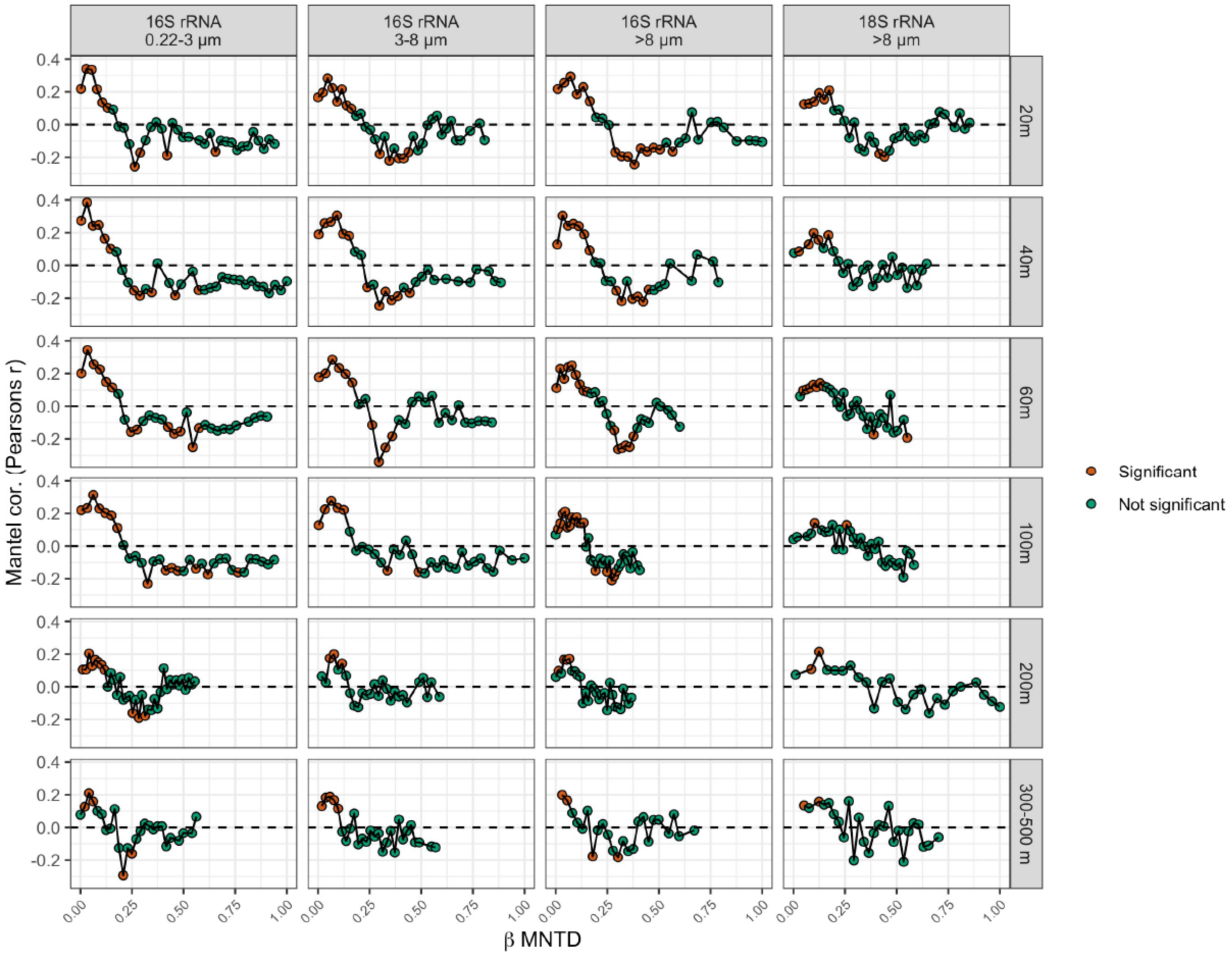

Figure S6: **Mantel-correlogram that shows linear correlation between bins of phylogenetic dissimilarity and habitat differences for 0.22-3 µm, 3-8 µm and >8 µm size fractions of the epi- and mesopelagic prokaryotic (16S rRNA) and eukaryotic (18S rRNA) communities.** Significant correlations are noted by red dots and non-significant by blue dots, respectively. Temperature was used to measure habitat differences and the βMNTI index to calculate phylogenetic dissimilarity. A significant positive correlation between habitat difference and phylogenetic dissimilarity of communities for smaller phylogenetic dissimilarities indicated phylogenetic signal, which was lost at larger dissimilarities in all data subsets.

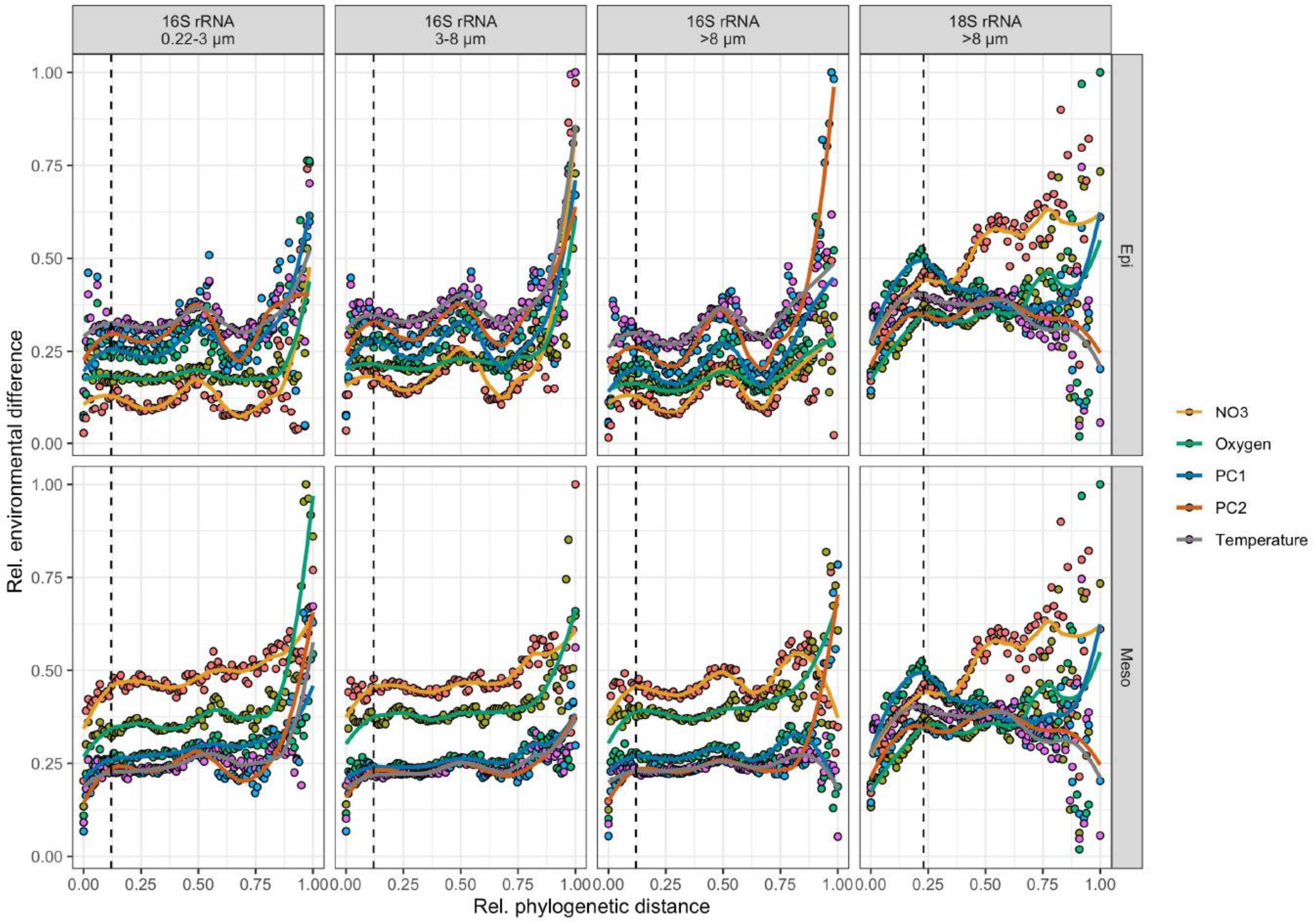

Figure S7: **Habitat differences between pairs of ASVs against their phylogenetic dissimilarity for 0.22-3 µm, 3-8 µm and >8 µm size fractions of the epi- and mesopelagic prokaryotic (16S rRNA) and eukaryotic (18S rRNA) communities.** Each point represents a median within a phylogenetic dissimilarity bin. Colour indicates the environmental parameter used for characterizing habitat preference of each ASV. The vertical dashed line displays the phylogenetic dissimilarity at which the curve fit is no longer linear, which indicates the loss of phylogenetic signal.

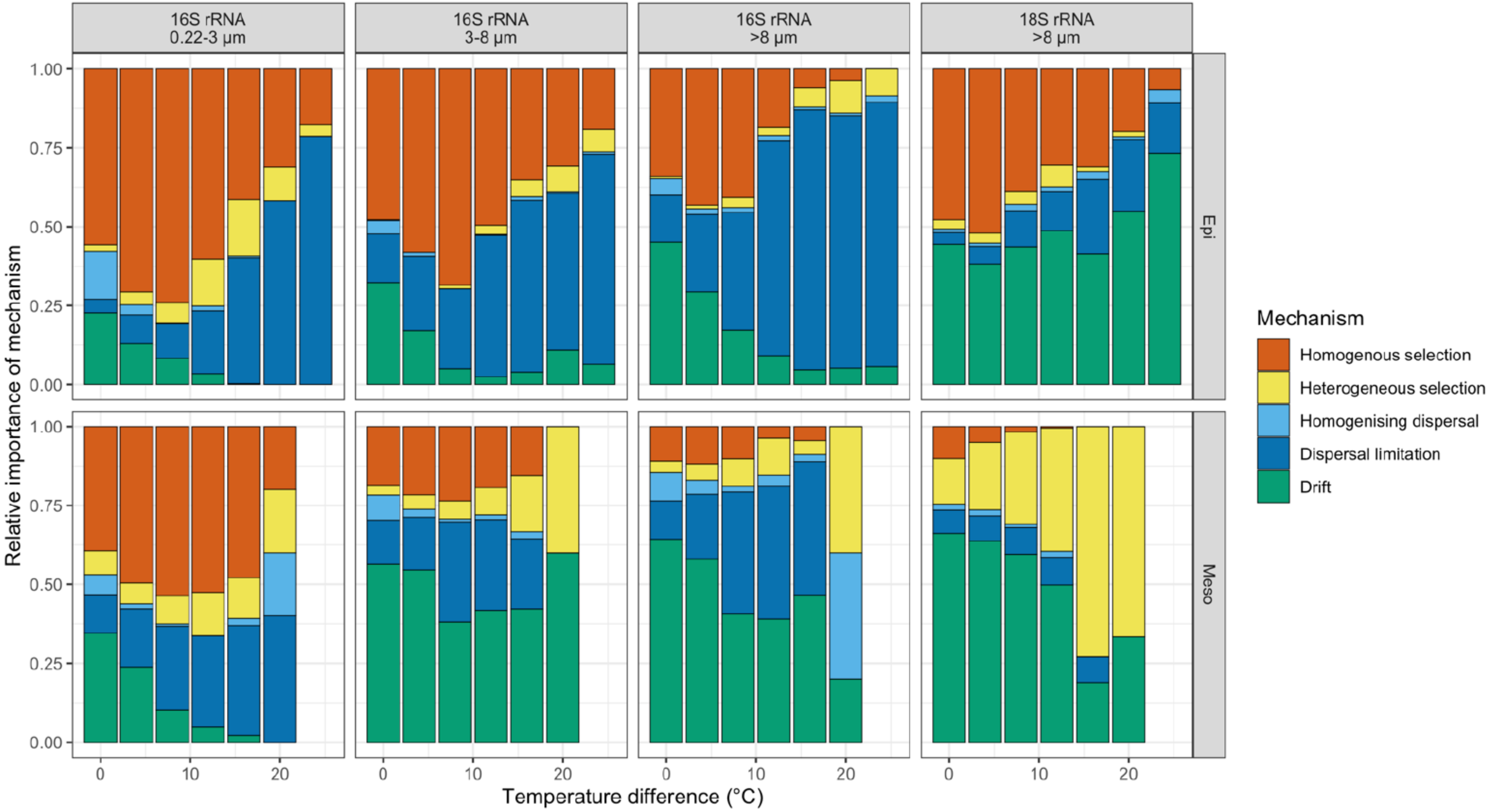

Figure S8: **Relative importance of five ecological mechanisms with increasing temperature difference between 0° and 28°C shaping biogeographic patterns of epi- and mesopelagic prokaryotic (16S rRNA) and eukaryotic (18S rRNA) communities as captured by different β-diversity metrics between the subantarctic and subarctic Pacific.** For prokaryotes size fractions 0.22-3 µm, 3-8 µm and >8 µm are shown. Temperature differences from 0-4°, 4-8°, 8-12°, 12-16°, 16-20°, 20-24 and 24-28°C are pooled and shown as one bar.

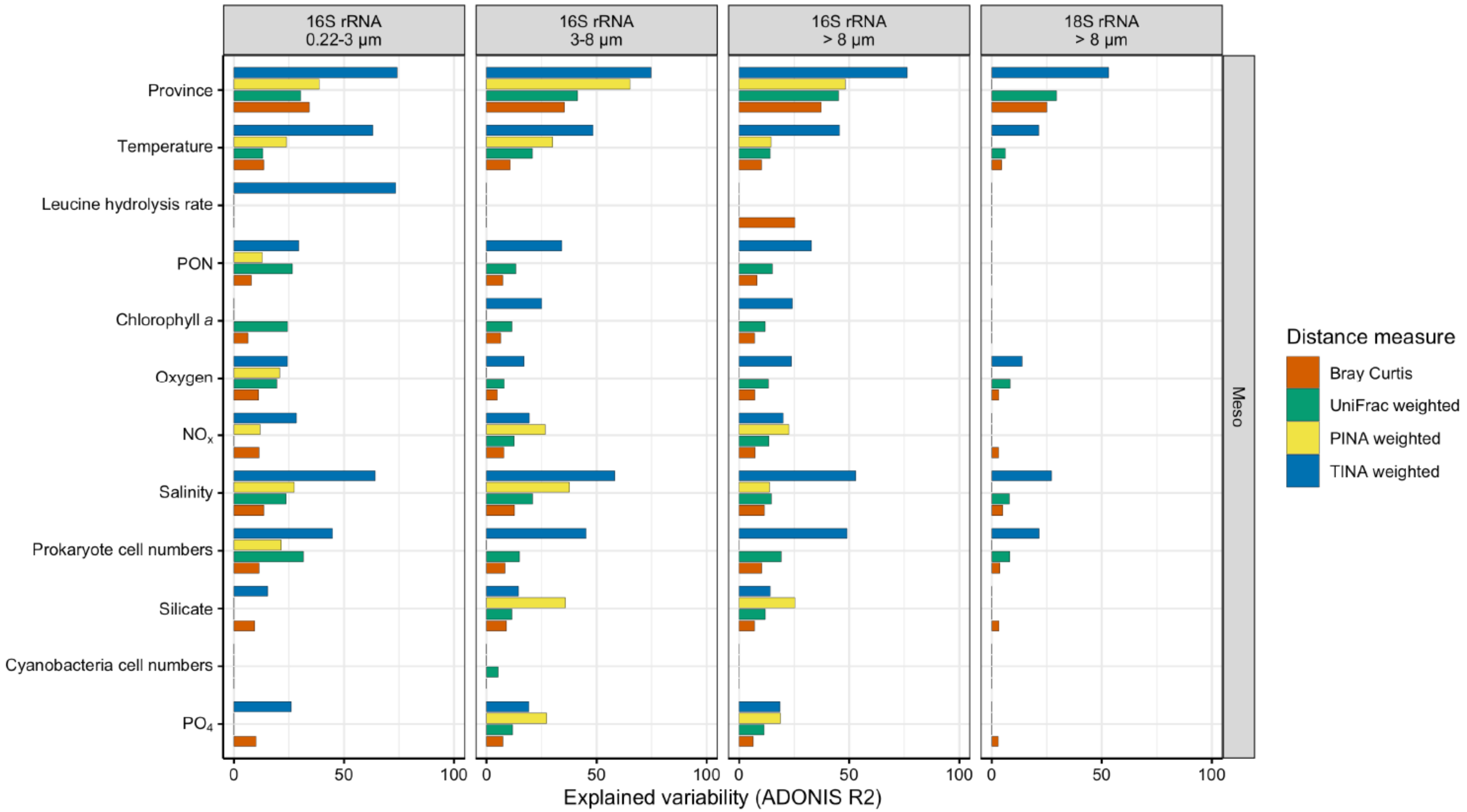

Figure S9: **Community variance individually explained by single environmental variables for different dissimilarity indices (Bray-Curtis, UniFrac, TINA and PINA) for FL, SPA and LPA prokaryotic communities in mesopelagic depths.** Explained variance was inferred from PERMANOVA and only significant results with Benjamini-Hochberg adjusted p-values < 0.05 are displayed.
